# Obstructive Sleep Apnea Syndrome Exacerbates NASH Progression via Selective Autophagy‐Mediated Eepd1 Degradation

**DOI:** 10.1002/advs.202405955

**Published:** 2024-06-25

**Authors:** Jie Xiong, Ying Xu, Ning Wang, Shengming Wang, Yao Zhang, Sijia Lu, Xiaoman Zhang, Xingxing Liang, Chuchu Liu, Quanxin Jiang, Junting Xu, Qiqi Qian, Peihui Zhou, Limin Yin, Feng Liu, Suzhen Chen, Shankai Yin, Junli Liu

**Affiliations:** ^1^ Shanghai Diabetes Institute Department of Endocrinology and Metabolism Shanghai Sixth People's Hospital Affiliated to Shanghai Jiao Tong University School of Medicine Shanghai 200233 China; ^2^ Department of Otolaryngology Head and Neck Surgery & Shanghai Key Laboratory of Sleep Disordered Breathing & Otolaryngology Institute of Shanghai Jiao Tong University Shanghai Sixth People's Hospital Affiliated to Shanghai Jiao Tong University School of Medicine Shanghai 200233 China; ^3^ Lingang Laboratory Shanghai 200031 China

**Keywords:** autophagic degradation, CIH, DNA repair enzyme, nonalcoholic steatohepatitis, retigabine dihydrochloride

## Abstract

Obstructive sleep apnea syndrome (OSAS), characterized by chronic intermittent hypoxia (CIH), is an independent risk factor for aggravating non‐alcoholic steatohepatitis (NASH). The prevailing mouse model employed in CIH research is inadequate for the comprehensive exploration of the impact of CIH on NASH development due to reduced food intake observed in CIH‐exposed mice, which deviates from human responses. To address this issue, a pair‐feeding investigation with CIH‐exposed and normoxia‐exposed mice is conducted. It is revealed that CIH exposure aggravates DNA damage, leading to hepatic fibrosis and inflammation. The analysis of genome‐wide association study (GWAS) data also discloses the association between Eepd1, a DNA repair enzyme, and OSAS. Furthermore, it is revealed that CIH triggered selective autophagy, leading to the autophagic degradation of Eepd1, thereby exacerbating DNA damage in hepatocytes. Notably, *Eepd1* liver‐specific knockout mice exhibit aggravated hepatic DNA damage and further progression of NASH. To identify a therapeutic approach for CIH‐induced NASH, a drug screening is conducted and it is found that Retigabine dihydrochloride suppresses CIH‐mediated Eepd1 degradation, leading to alleviated DNA damage in hepatocytes. These findings imply that targeting CIH‐mediated Eepd1 degradation can be an adjunctive approach in the treatment of NASH exacerbated by OSAS.

## Graphical abstract

Chronic intermittent hypoxia increases the expression of Hif1α, and Hif1α promotes autophagy activation in hepatocytes, which accelerates the autophagic degradation of Eepd1, a DNA repair enzyme, and hence exacerbates DNA damage in hepatocytes. The increased DNA damage exacerbates liver inflammation, fibrosis, and NASH progression. Eepd1 liver‐specific knockout mice aggravate DNA damage and then exacerbate liver inflammation, fibrosis, and NASH progression. Retigabine dihydrochloride, targeting CIH‐mediated Eepd1 degradation, can represent an effective treatment for CIH‐induced NASH.

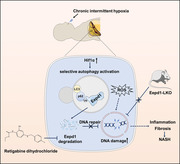

## 1 Introduction

OSAS is a prevalent sleep‐disordered breathing disorder characterized by CIH and fragmented sleep patterns, posing a substantial risk of fatal complications.^[^ ^1^ ^]^ Additionally, OSAS is associated with heightened morbidity and mortality due to various metabolic abnormalities, including cardiovascular complications, diabetes, cerebrovascular disorders, and multiple types of cancers.^[^ ^1^, ^2^ ^]^ Nonalcoholic fatty liver disease (NAFLD) is a metabolic disorder with a remarkable global prevalence that affects ≈25% of the population. It encompasses a spectrum of conditions ranging from NAFLD to the more severe manifestation of NASH, which is distinguished by necrotizing inflammation and expedited fibrosis.^[^ ^3^ ^]^ Numerous clinical investigations have consistently demonstrated a close association between OSAS and NAFLD/NASH.^[^ ^4^ ^]^ Moreover, CIH has been identified as an independent risk factor that exacerbates liver injury and promotes the progression from NAFLD to NASH.^[^ ^5^ ^]^ Additionally, the successful induction of a pediatric NASH model through CIH in juvenile mice further indicated that CIH could hasten NASH progression.^[^ ^6^ ^]^ Nevertheless, the precise underlying mechanism by which OSAS influences the development of NASH remains unclear.

Studies have documented that CIH can trigger the production of reactive oxygen species (ROS), thereby intensifying apnea and cardiovascular impairments. ROS have long been recognized as key mediators of DNA damage, and their involvement in such damage exhibits a multifaceted and diverse nature. For instance, ROS can cause double‐strand breaks in DNA by directly destroying the DNA sugar backbone with high‐energy impact, as well as induce oxidative stress‐related damage through the generation of free radicals within cells. Additionally, oxidative base modifications of DNA sequence can directly instigate other forms of DNA damage.^[^ ^7^ ^]^ Furthermore, DNA damage has been implicated in the exacerbation of the necrotizing inflammation and fibrosis characteristic of NASH progression.^[^ ^8^ ^]^ The confluence of inflammation, fibrosis, and heightened lipid accumulation collectively aggravates the transition from NAFLD to NASH.

DNA damage triggers a cascade of cellular self‐rescue mechanisms collectively known as the DNA damage response. Examples of these mechanisms include homologous recombination (HR), nonhomologous end joining, base excision repair, and nucleotide excision repair. HR, for instance, governs the repair of DNA double‐strand breaks and interstrand crosslinks through an intricate network of processes. Key enzymes involved in HR, including Eepd1, Mus81, Gen1, DNA2, and BLM, play vital roles in safeguarding the integrity and fidelity of our DNA sequence.^[^ ^9^ ^]^ However, the impact of CIH on these HR enzymes during the progression of NASH remains unknown.

Clinical studies have established a link between obesity and OSAS, with the prevalence of OSAS escalating alongside increased appetite and body weight.^[^ ^10^ ^]^ Numerous studies have employed murine models to investigate CIH‐induced NASH.^[^ ^6^, ^11^ ^]^ It is noteworthy that the existing murine models display reduced appetite under CIH conditions, leading to reduced food intake and lower body weight when both CIH‐exposed and control mice have unrestricted access to food.^[^ ^11^ ^]^ Considering the significant influence of dietary factors and weight fluctuations on liver pathology, we believe that the disparity in food intake between mice and humans renders the current murine model inadequate for studying the direct influence of CIH on the development of NASH unless substantial modifications are made to the experimental design.

In this study, we conducted a pair‐feeding study with the CIH‐exposed mice and normoxia‐exposed mice to investigate the direct effect of CIH on NASH development. Our findings revealed that CIH instigated a distinctive form of selective autophagy, which promoted the autophagic degradation of Eepd1, a DNA repair enzyme, and consequently aggravated DNA damage in hepatocytes. The enhanced DNA damage further aggravated liver inflammation, fibrosis, and the overall progression of NASH.

## 2 Results

### 2.1 CIH Exacerbates NASH Progression in Mice

**Figure 1:**
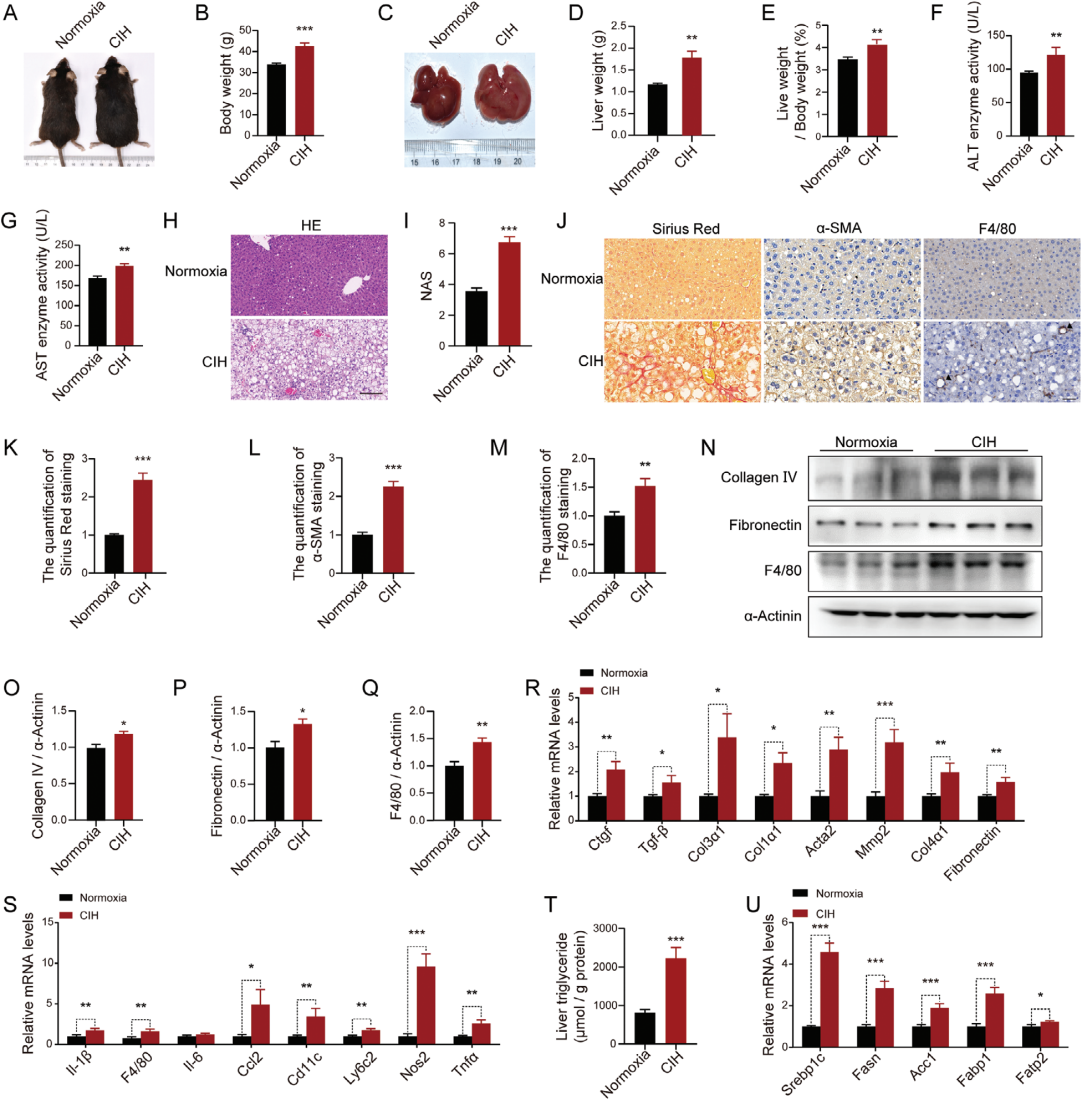
CIH exacerbates NASH progression in vivo. After 4 weeks of being fed HFD, male mice were subjected to CIH or normoxia conditions while continuing on the HFD. A) Representative images of HFD‐fed mice exposed to CIH (10 weeks) or normoxia. B) Body weight of HFD‐fed mice exposed to CIH (10 weeks) or normoxia (*n* = 10). C) Representative images of liver morphology from HFD‐fed mice exposed to CIH (10 weeks) or normoxia. D) Liver weight of HFD‐fed mice exposed to CIH (10 weeks) or normoxia (*n* = 10). E) Liver weight to body weight ratios of HFD‐fed mice exposed to CIH (10 weeks) or normoxia (*n* = 10). F) Serum ALT concentrations of HFD‐fed mice exposed to CIH (10 weeks) or normoxia (*n* = 10). G) Serum AST concentrations of HFD‐fed mice exposed to CIH (10 weeks) or normoxia (*n* = 10). H) H&E staining of liver sections from HFD‐fed mice exposed to CIH (10 weeks) or normoxia. Scale bar, 50 µm. I) NAFLD activity score (NAS) of HFD‐fed mice exposed to CIH (10 weeks) or normoxia. J) Representative Sirius Red staining and immunohistochemical staining (α‐SMA, F4/80) of liver sections from HFD‐fed mice exposed to CIH (10 weeks) or normoxia. Scale bar, 50 µm. The black arrow represent a crown‐like structure. K) Quantitative analysis of Sirius Red staining from (J). L) Quantitative analysis of α‐SMA staining from (J). M) Quantitative analysis of F4/80 staining from (J). N) The protein levels of Collagen IV, Fibronectin, and F4/80 in the livers of HFD‐fed mice exposed to CIH (10 weeks) or normoxia (*n* = 3 per group). O) Quantitative analysis of the Collagen IV protein levels from (N). P) Quantitative analysis of the Fibronectin protein levels from (N). Q) Quantitative analysis of the F4/80 protein levels from (N). R) The mRNA levels of genes involved in fibrosis in the livers of HFD‐fed mice exposed to CIH (10 weeks) or normoxia. S) The mRNA levels of inflammatory genes in the livers of HFD‐fed mice exposed to CIH (10 weeks) or normoxia. T) Liver triglyceride concentrations of HFD‐fed mice exposed to CIH (10 weeks) or normoxia (*n* = 10). U) The mRNA levels of lipid metabolism‐related genes in the livers of HFD‐fed mice exposed to CIH (10 weeks) or normoxia. Data are presented as mean ± S.E.M. Significance was assessed by Student's *t*‐test (B, D, E, F, G, I, K, L, M, O, Q, T, U) or Mann‐Whitney *U* test (P, R, S). ^*^ *p *< 0.05, ^**^ *p *< 0.01, ^***^ *p *< 0.001 versus control.

To thoroughly investigate the impact of CIH on the progression of NASH, we exposed diet‐induced obese mice to either CIH or normoxia conditions for 10 weeks. The mice had ad libitum access to a high‐fat diet (HFD) throughout the CIH treatment. Consistent with previous findings,^[^ ^11^ ^]^ mice in the CIH group exhibited a significant decrease in food intake and body weight (Figure S1A,B, Supporting Information). It is worth noting that CIH exerts its influence on the central nervous system of mice by altering food intake, which in turn indirectly impacts peripheral organs like the liver.^[^ ^12^ ^]^ To eliminate interruptions caused by differences in food intake and to elucidate the direct impact of CIH on NASH progression, we designed a pair‐feeding experiment in which HFD‐fed mice, receiving the same amount of diet, were exposed to either CIH or normoxia conditions. Following 10 weeks of adaptation to CIH, the mice displayed higher body weights compared to the normoxia controls (**Figure** **1**A,B). The liver weights and liver‐to‐body weight ratios of CIH mice were also higher than those of the normoxia mice (Figure 1C–E). Notably, CIH‐exposed mice exhibited more pronounced liver injury compared to the normoxia group, as evidenced by elevated serum levels of alanine aminotransferase (ALT) (Figure 1F) and aspartate aminotransferase (AST) (Figure 1G), along with upregulated AST to ALT ratios (Figure S1C, Supporting Information). Moreover, hematoxylin and eosin (H&E) staining revealed increased steatosis in the livers of CIH‐exposed mice (Figure 1H). Consistently, the NAFLD Activity Score (NAS), based on the assessment of H&E staining, was higher in the CIH‐exposed group compared to the control group (Figure 1I; Figure S1D, Supporting Information). Furthermore, Sirius Red staining revealed increased collagen deposition in the livers of CIH‐exposed mice compared to the normoxia group (Figure 1J,K). Immunohistochemical (IHC) staining of the fibrosis‐associated protein, alpha‐smooth muscle actin (α‐SMA) (Figure 1J,L) and immunoblot analysis of the fibrosis‐associated protein, Collagen IV and Fibronectin (Figure 1N–P), confirmed the presence of more severe fibrosis levels in the CIH group. Consistent with these observations, CIH‐exposed mice demonstrated upregulated mRNA expression of genes associated with fibrosis (Figure 1R). Moreover, mice subjected to CIH exhibited heightened inflammatory responses compared to the normoxia group, as indicated by IHC staining (Figure 1J,M) and immunoblot analysis of F4/80 (Figure 1N,Q), as well as mRNA expression levels of cytokines in the liver (Figure 1S). Additionally, the CIH group displayed significantly elevated lipid accumulation as revealed by hepatic TG analysis (Figure 1T) and expression of genes involved in fatty acid uptake and synthesis (Figure 1U). We also performed similar CIH exposure experiments with C57BL/6J mice fed with a normal chow (NC) diet. CIH exposure led to mild liver damage in normal‐fed mice but did not progress to the extent of NASH (Figure S1E–Z, Supporting Information).

Collectively, our findings from the CIH‐exposed mice with pair‐feeding treatment demonstrate that CIH substantially accelerates the progression of NASH.

### 2.2 CIH Aggravates Fibrosis, Inflammation, and Lipid Accumulation in Hepatocytes

**Figure 2:**
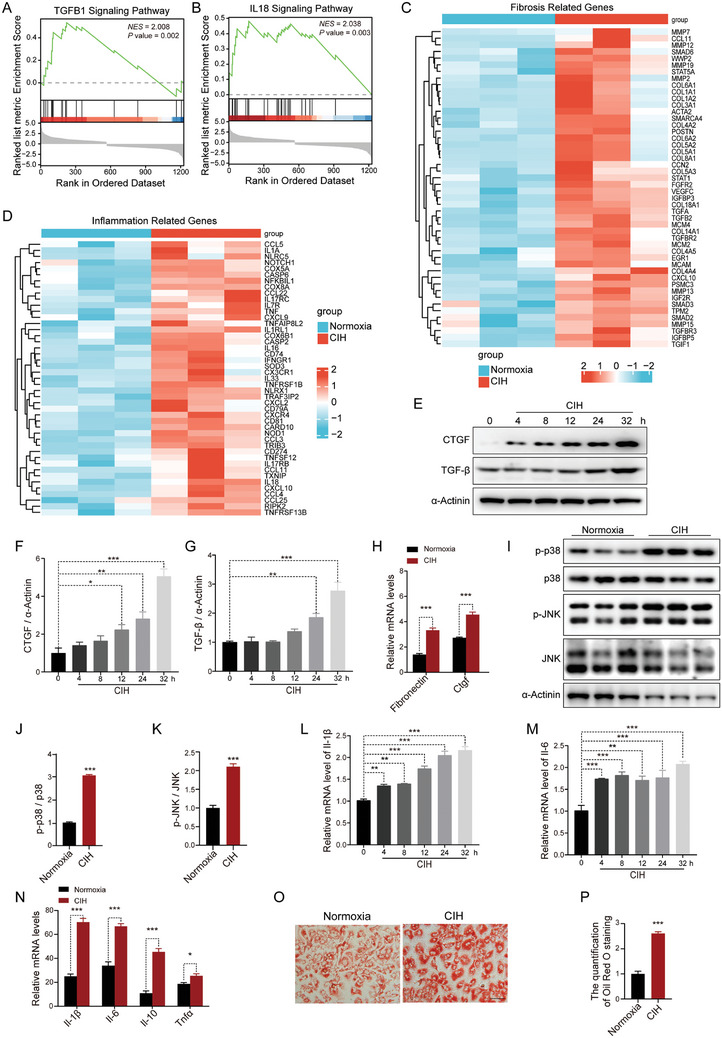
CIH aggravates fibrosis, inflammation, and lipid accumulation in hepatocytes with PAOA stimulation. A) Statistically significant probable relevant fibrosis signaling pathways analyzed by GSEA plots of RNA‐seq data obtained from livers of HFD‐fed mice exposed to CIH (10 weeks) or normoxia. *NES* for normalized enrichment score. B) Statistically significant probable relevant inflammatory signaling pathways analyzed by GSEA plots of RNA‐seq data obtained from livers of HFD‐fed mice exposed to CIH (10 weeks) or normoxia. *NES* for normalized enrichment score. C) Differentially expressed fibrosis‐related genes in RNA‐seq data obtained from livers of HFD‐fed mice exposed to CIH (10 weeks) or normoxia. D) Differentially expressed inflammation‐related genes in RNA‐seq data obtained from livers of HFD‐fed mice exposed to CIH (10 weeks) or normoxia. E) The protein levels of CTGF and TGF‐β in the primary hepatocytes subjected to CIH treated with PAOA (450 µm, 32 h). F) Quantitative analysis of CTGF protein levels performed utilizing the three independent Western Blot results presented in panels (E), Figure S2B,C (Supporting Information). G) Quantitative analysis of TGF‐β protein levels performed utilizing the three independent Western Blot results presented in panels (E), Figure S2B,C (Supporting Information). H) The mRNA levels of genes involved in fibrosis in the primary hepatocytes treated with PAOA (450 µm, 32 h) under CIH (32 h) or normoxia condition. I) The protein levels of p‐p38 and p‐JNK in the primary hepatocytes treated with PAOA (450 µm, 32 h) under CIH (32 h) or normoxia condition (*n* = 3 per group). J) Quantitative analysis of p‐p38/p38 ratio from (I). K) Quantitative analysis of p‐JNK/JNK ratio from (I). L) The mRNA levels of *Il‐1β* in the primary hepatocytes subjected to CIH. M) The mRNA levels of *Il‐6* in the primary hepatocytes subjected to CIH. N) The mRNA levels of inflammatory genes in the primary hepatocytes treated with PAOA (450 µm, 32 h) under CIH (32 h) or normoxia condition. O) The Oil Red O staining in the primary hepatocytes treated with PAOA (450 µm, 32 h) under CIH (32 h) or normoxia condition. Scale bar, 50 µm. P) Quantitative analysis of Oil Red O staining from (O). Data are presented as mean ± S.E.M. Significance was assessed by Student's *t*‐test (H, J, K, N, P) or one‐way ANOVA (F, G, L, M). ^*^ *p *< 0.05, ^**^ *p *< 0.01, ^***^ *p *< 0.001 versus control.

Next, we conducted RNA‐seq analysis on liver samples from mice exposed to CIH. RNA‐seq analysis revealed significant alterations in gene expression, with 2162 genes down‐regulated and 2237 genes up‐regulated in the CIH group compared to the control group (Log_2_ Fold change >1 or <−1) (Figure S2A, Supporting Information). Inflammatory and fibrosis signaling pathways were the enriched biological process terms (False Discovery Rate (FDR) <0.05), according to Gene Set Enrichment Analysis (GSEA) (**Figure** **2**A,B). Consistently, RNA‐seq analysis demonstrated the upregulation of genes associated with inflammation and fibrosis in the CIH group (Figure 2C,D).

To further explore the direct impact of CIH on hepatocytes, we cultured primary mouse hepatocytes in a CIH chamber, with or without palmitic acid and oleic acid (PAOA) treatment. Our analysis uncovered a time‐dependent increase in two pro‐fibrosis factors, CTGF and TGF‐β, resulting from exposure to CIH (Figure 2E–G; Figure S2B,C, Supporting Information). Moreover, we observed a significant upregulation of mRNA levels of liver fibrosis‐related genes in CIH‐exposed hepatocytes with or without PAOA treatment (Figure 2H; Figure S2D, Supporting Information). CIH exposure also led to increased phosphorylation of p38 and JNK, indicating activation of inflammation pathways (Figure 2I–K; Figure S2E–G, Supporting Information). Similarly, the mRNA levels of inflammatory cytokines were elevated under CIH conditions (Figure 2L–N; Figure S2H, Supporting Information). We also observed increased lipid accumulation in hepatocytes under CIH conditions, as evidenced by Oil Red O staining (Figure 2O–P; Figure S2I,J, Supporting Information). Collectively, these findings demonstrate that CIH can directly promote inflammation and fibrosis in hepatocytes, ultimately leading to the development of liver pathology.

### 2.3 CIH Leads to the Occurrence of NASH by Inducing DNA Damage

**Figure 3:**
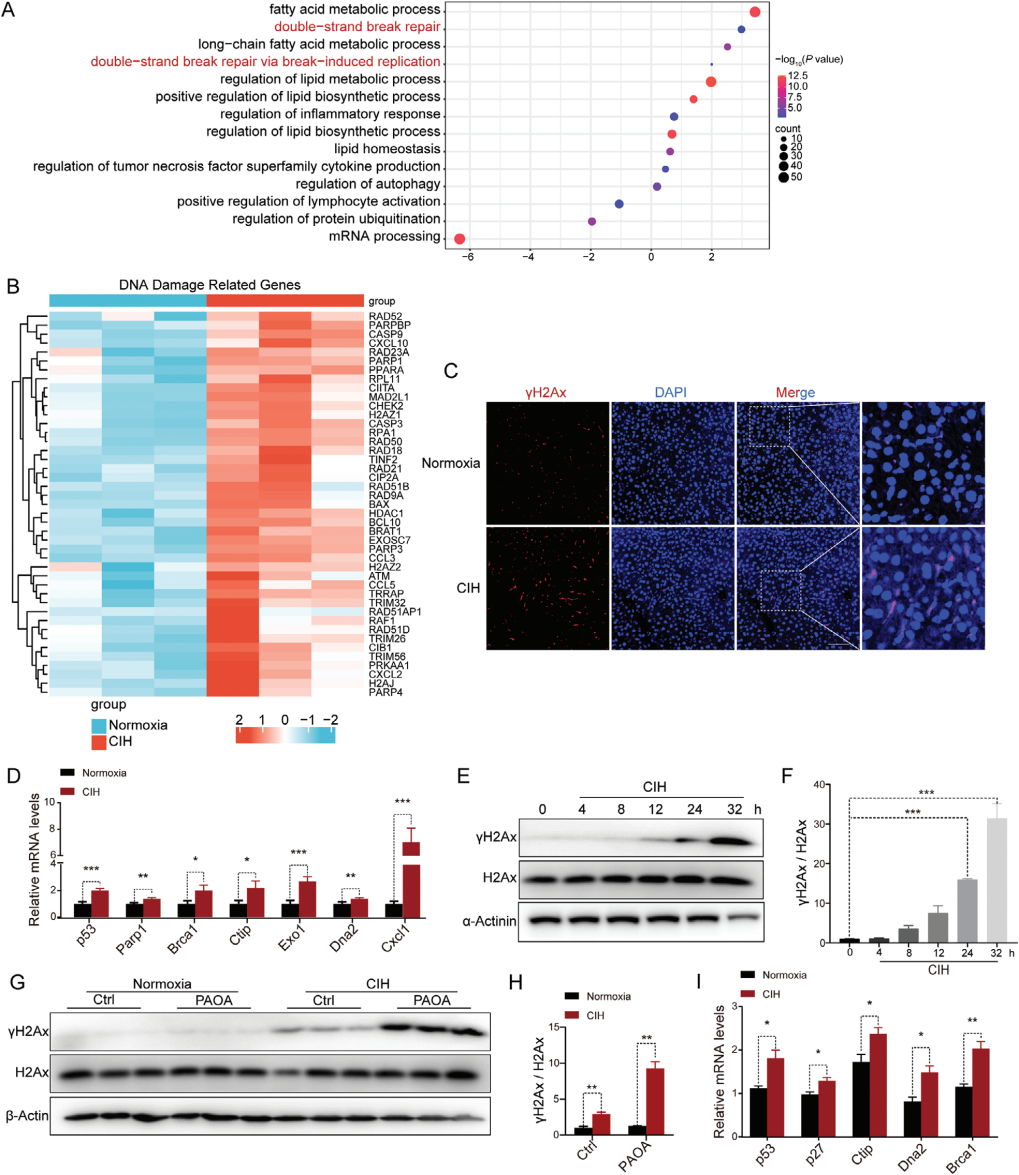
CIH exacerbates DNA damage. A) The Ingenuity Pathway Analysis of RNA‐seq data obtained from livers of HFD‐fed mice exposed to CIH (10 weeks) or normoxia. B) Differentially expressed DNA damage‐related genes in RNA‐seq data obtained from livers of HFD‐fed mice exposed to CIH (10 weeks) or normoxia. C) Immunofluorescence of γH2Ax of liver sections from HFD‐fed mice exposed to CIH (10 weeks) or normoxia. Nuclei were counterstained with DAPI. Scale bar, 50 µm. D) The mRNA levels of DNA damage‐related genes in the liver of HFD‐fed mice exposed to CIH (10 weeks) or normoxia. E) The protein levels of γH2Ax in the primary hepatocytes subjected to CIH. F) Quantitative analysis of γH2Ax/H2Ax ratio performed utilizing the three independent Western Blot results presented in panels (E), Figure S3E,F (Supporting Information). G) The protein levels of γH2Ax in the primary hepatocytes treated with PAOA (450 µm, 32 h) under CIH (32 h) or normoxia condition (*n* = 3 per group). H) Quantitative analysis of γH2Ax/H2Ax ratio from (G). I) The mRNA levels of DNA damage‐related genes in the primary hepatocytes treated with PAOA (450 µm, 32 h) under CIH (32 h) or normoxia condition. Data are presented as mean ± S.E.M. Significance was assessed by one‐way ANOVA (F), Student's *t‐*test (H, I), or Mann–Whitney *U* test (D). *p < 0.05, **p < 0.01, ***p < 0.001 versus control.

To investigate the underlying mechanisms by which CIH exacerbates NASH progression, we re‐analyzed the RNA‐seq data by Gene Ontology analysis. Notably, our analysis revealed that the pathways associated with DNA damage were significantly enriched (FDR<0.05) (**Figure** **3**A). Consistently, the RNA‐seq results demonstrated a significant upregulation of DNA damage marker genes in the liver of mice exposed to CIH (Figure 3B). To validate these findings, we examined mouse liver samples with immunofluorescence (IF) staining and observed a substantial increase in the expression of γH2Ax, a marker for DNA double‐strand breaks, in the livers of CIH‐exposed mice (Figure 3C; Figure S3A, Supporting Information). Moreover, the mRNA expression levels of other DNA damage‐related genes were significantly upregulated (Figure 3D). Consistently, CIH exposure also aggravated the DNA damage in NC‐fed mice as determined by IF (Figure S3B,C, Supporting Information) and qRT‐PCR analysis (Figure S3D, Supporting Information). Next, we validated these findings in cellular experiments. Immunoblot analysis revealed that CIH exposure led to a time‐dependent increase in the expression of γH2Ax protein in primary hepatocytes (Figure 3E,F; Figure S3E,F, Supporting Information). PAOA stimulation further upregulated the expression of γH2Ax in CIH‐exposed hepatocytes (Figure 3G,H). Additionally, the upregulation of mRNA levels of other DNA damage‐related genes also indicated that CIH exacerbated DNA damage in hepatocytes with or without PAOA treatment (Figure 3I; Figure S3G, Supporting Information). Taken together, our findings suggest that CIH exacerbates DNA damage in hepatocytes, both in vivo and in vitro.

### 2.4 CIH‐Induced Downregulation of Eepd1 Exacerbates DNA Damage

**Figure 4:**
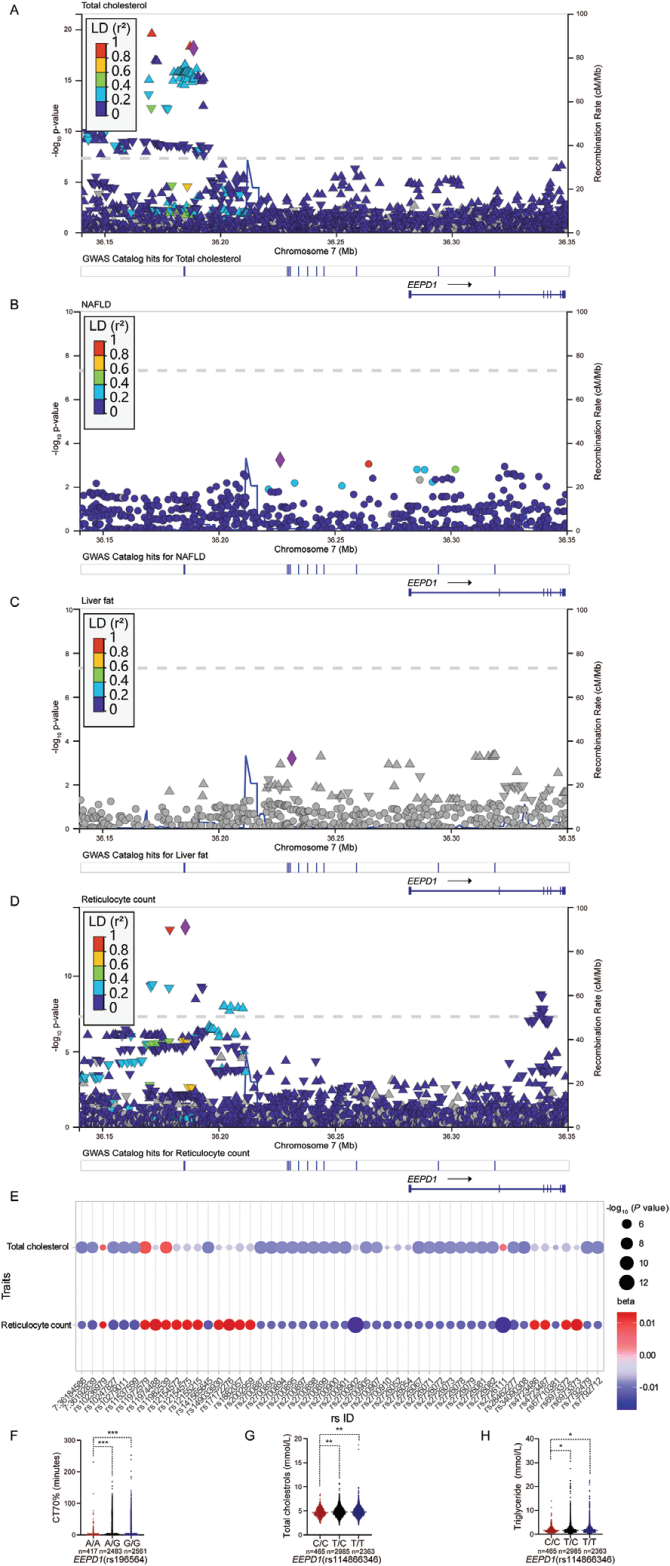
The association between EEPD1 with metabolic and OSAS‐related traits. A) The region plot shows the association between *EEPD1* genetic variation (rs34578041) with total cholesterol. B) The region plot shows the association between *EEPD1* genetic variation (rs117240369) with NAFLD. C) Region plot shows the association between *EEPD1* genetic variation (rs551923736) with liver fat. D) Region plot shows the association between *EEPD1* genetic variation (rs2700902) with reticulocyte count. E) The SNPs correlated with both “total cholesterol levels” and “reticulocyte count” within the front region of the *EEPD1* gene. F) Association between *Eepd1* (rs196564; A/A, wild‐type; A/G, heterozygous; G/G, mutation) with CT70% (cumulative time percentage of SpO_2_ < 70%). G) Association between *EEPD1* (rs114866346; C/C, wild‐type; T/C, heterozygous; T/T, mutation) with total cholesterol. H) Association between *EEPD1* (rs114866346; C/C, wild‐type; T/C, heterozygous; T/T, mutation) with triglyceride.

Accumulated evidence indicates that hypoxia‐induced ROS accumulation and inflammation response contribute to DNA damage. Physiologically, DNA damage promptly initiates the activation of DNA damage repair, an intrinsic cellular self‐rescue process. To investigate the impact of CIH on the DNA damage repair process, we conducted a systematic data mining of extant genetic research endeavors with the GWAS database from EMBL's (European Molecular Biology Laboratory) European Bioinformatics Institute (ftp.ebi.ac.uk). We discovered that *EEPD1*, an important DNA repair enzyme, has a potential relationship with both metabolic and hypoxia‐related traits (i.e., reticulocyte count) within the population.^[^ ^13^ ^]^ The outcomes underscored that the genetic polymorphisms of *EEPD1* exhibit robust genome‐wide significant (*p* < 5E‐8) associations with a spectrum of metabolic traits, encompassing total cholesterol levels (GWAS Catalog Accession: GCST90239673),^[^ ^14^ ^]^ LDL cholesterol levels, and HDL cholesterol levels (**Figure** **4**A; Table S1, Supporting Information).^[^ ^15^ ^]^ Meanwhile, The *EEPD1* genetic variations rs117240369 and rs551923736 have also been proposed to be significantly associated with NAFLD (GWAS Catalog Accession: GCST90054782)^[^ ^16^ ^]^ and liver fat (GWAS Catalog Accession: GCST90016673)^[^ ^17^ ^]^ respectively (Figure 4B,C; Table S2, Supporting Information). Additionally, the *EEPD1* polymorphisms demonstrated similarly significant correlations with OSAS‐related traits, notably reticulocyte count (GWAS Catalog Accession: GCST90002385)^[^ ^18^ ^]^ and red cell distribution width at genome‐wide significance (Figure 4D; and Table S1, Supporting Information).^[^ ^18^, ^19^ ^]^ Of note, the variant rs34578041 exhibited significant correlations with both total cholesterol levels and red cell distribution width (Table S1, Supporting Information). In addition, we observed a notable concentration of SNPs highly correlated with both “total cholesterol levels” and “reticulocyte count” within the front region of the *EEPD1* gene. Upon comparison, we identified 50 SNPs displaying significant associations with both traits, of which 37 exhibited concordant effects on both traits (Figure 4E). This observation suggests the potential presence of a genetic synergy or a shared genetic regulatory mechanism between these two traits.

In further pursuit of elucidating the association between *EEPD1* and OSAS, we conducted an analysis of GWAS data from 5773 individuals of OSAS patients from the ongoing SSHS (Shanghai Sleep Health Study) cohort.^[^ ^20^ ^]^ Intermittent hypoxia caused by respiratory pauses or hypoventilation during sleep is a significant pathological feature of OSAS. The CT70 index quantifies the severity of this feature, representing the proportion of sleep time during which blood oxygen saturation falls below 70% because of respiratory pauses, thereby indicating the extent of oxygen desaturation relative to total sleep duration. As shown in Figure 4F, individuals carrying the *EEPD1*, A/G, and G/G polymorphism demonstrated elevated CT70 values, typically indicating a more severe OSAS condition. Intriguingly, our findings also revealed a close correlation between total cholesterol and triglyceride levels with *EEPD1* in OSAS patients (Figure 4G,H).

**Figure 5:**
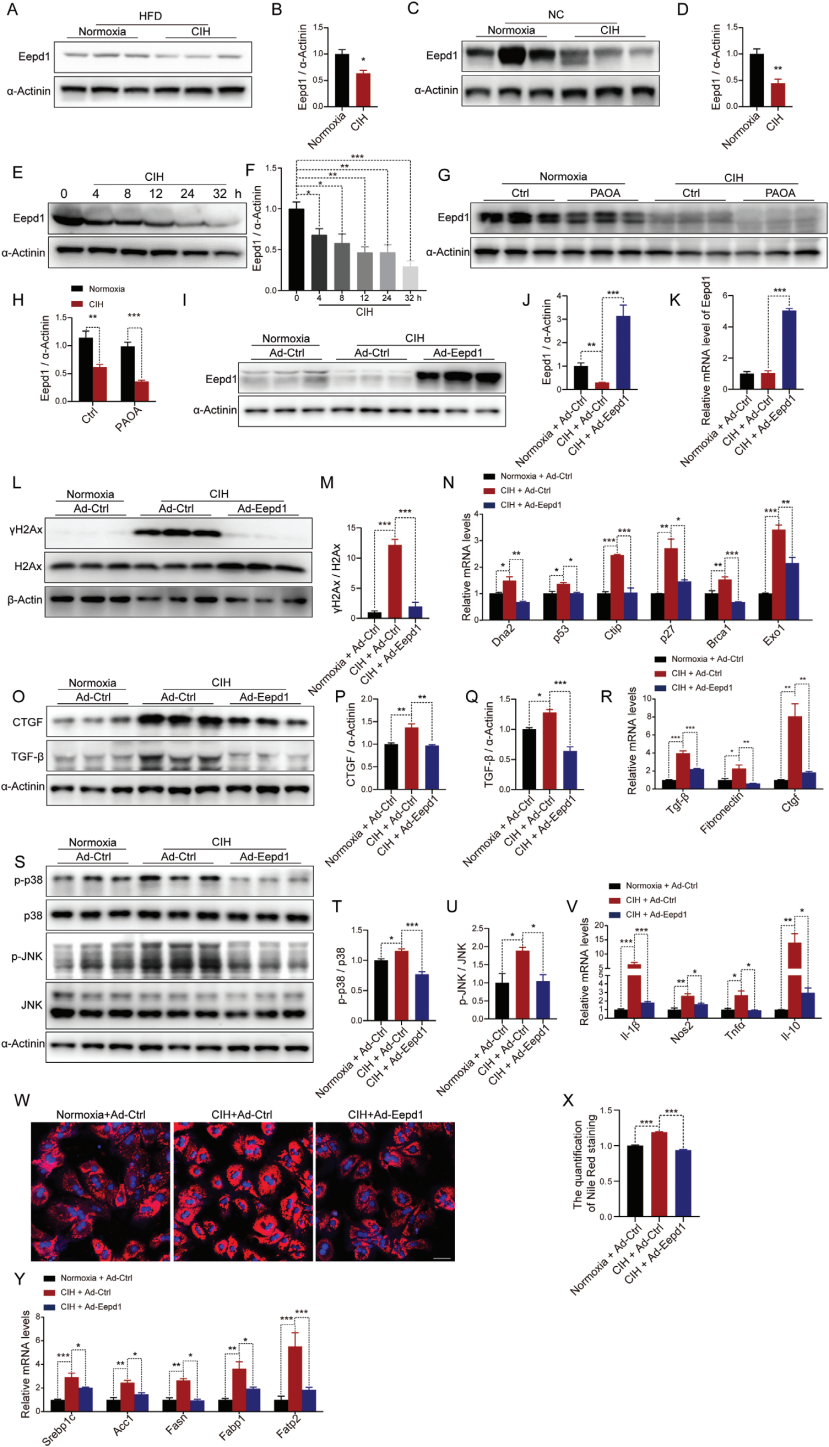
CIH leads to the degradation of Eepd1 and Eepd1 overexpression alleviates CIH‐induced NASH. A) The protein levels of Eepd1 in the livers of HFD‐fed mice exposed to CIH (10 weeks) or normoxia (*n* = 3 per group). B) Quantitative analysis of the protein levels of Eepd1 from (A). C) The protein levels of Eepd1 in the livers of NC‐fed mice exposed to CIH (10 weeks) or normoxia (*n* = 3 per group). D) Quantitative analysis of the protein levels of Eepd1 from (C). E) The protein levels of Eepd1 in the primary hepatocytes subjected to CIH. F) Quantitative analysis of the Eepd1 protein levels performed utilizing the three independent Western Blot results presented in panels (E), Figure S4A,B (Supporting Information). G) The protein levels of Eepd1 in the primary hepatocytes treated with PAOA (450 µm, 32 h) under CIH (32 h) or normoxia condition (*n* = 3 per group). (H) Quantitative analysis of the protein levels of Eepd1 from (G). I) The protein levels of Eepd1 in the primary hepatocytes overexpressed *Eepd1* treated with PAOA (450 µm, 32 h) under CIH (32 h) or normoxia condition (*n* = 3 per group). J) Quantitative analysis of the protein levels of Eepd1 from (I). K) The mRNA levels of *Eepd1* in the primary hepatocytes overexpressed *Eepd1* treated with PAOA (450 µm, 32 h) under CIH (32 h) or normoxia condition. L) The protein levels of γH2Ax in the primary hepatocytes overexpressed *Eepd1* treated with PAOA (450 µm, 32 h) under CIH (32 h) or normoxia condition (*n* = 3 per group). M) Quantitative analysis of γH2Ax/H2Ax ratio from (L). N) The mRNA levels of DNA damage‐related genes in the primary hepatocytes overexpressed *Eepd1* treated with PAOA (450 µm, 32 h) under CIH (32 h) or normoxia condition. O) The protein levels of CTGF and TGF‐β in the primary hepatocytes overexpressed *Eepd1* treated with PAOA (450 µm, 32 h) under CIH (32 h) or normoxia condition (*n* = 3 per group). P) Quantitative analysis of the CTGF protein levels from (O). Q) Quantitative analysis of the TGF‐β protein levels from (O). R) The mRNA levels of genes involved in fibrosis in the primary hepatocytes overexpressed *Eepd1* treated with PAOA (450 µm, 32 h) under CIH (32 h) or normoxia condition. S) The protein levels of p‐p38 and p‐JNK in the primary hepatocytes overexpressed *Eepd1* treated with PAOA (450 µm, 32 h) under CIH (32 h) or normoxia condition (*n* = 3 per group). T) Quantitative analysis of p‐p38/p38 ratio from (S). (U) Quantitative analysis of p‐JNK/JNK ratio from (S). V) The mRNA levels of inflammatory genes in the primary hepatocytes overexpressed *Eepd1* treated with PAOA (450 µm, 32 h) under CIH (32 h) or normoxia condition. (W) The Nile Red staining in the primary hepatocytes overexpressed *Eepd1* treated with PAOA (450 µm, 32 h) under CIH (32 h) or normoxia condition. X) Quantitative analysis of Nile Red staining from (W). Y) The mRNA levels of lipid metabolism‐related genes in the primary hepatocytes overexpressed *Eepd1* treated with PAOA (450 µm, 32 h) under CIH (32 h) or normoxia condition. Data are presented as mean ± S.E.M. Significance was assessed by Student's *t‐*test (B, D, H), one‐way ANOVA (F, J, K, M, N, P, Q, R, T, U, V, X) or Kruskal Wallis test (Y). *p < 0.05, **p < 0.01, *** *p *< 0.001 versus control.

Further corroborating this, immunoblot analysis demonstrated that CIH exposure resulted in reduced Eepd1 levels in the livers of both HFD‐fed and NC‐fed mice (**Figure** **5**A–D). In vitro analysis also revealed a decrease in Eepd1 protein levels with prolonged CIH exposure (Figure 5E,F; Figure S4A,B, Supporting Information). PAOA stimulation further enhanced the CIH‐induced reduction in Eepd1 levels (Figure 5G,H). Moreover, we observed a negative correlation between Eepd1 levels and the extent of DNA damage (Figure S4C, Supporting Information), fibrosis (Figure S4D, Supporting Information), and inflammation (Figure S4E, Supporting Information) in the livers of mice under normoxia or CIH conditions. To further investigate the role of Eepd1 in CIH‐induced NASH, we conducted an overexpression study in primary hepatocytes exposed to CIH (Figure 5I–K). Eepd1 overexpression reduced DNA damage under CIH conditions (Figure 5L–N), consistent with previous reports of Eepd1's DNA repair capabilities.^[^ ^9^, ^21^ ^]^ Remarkably, our results demonstrated that Eepd1 overexpression significantly inhibited the CIH‐induced upregulation of pro‐fibrotic factors (Figure 5O–R). Additionally, Eepd1 overexpression decreased the phosphorylation of p38 and JNK, as well as the levels of CIH‐elevated inflammatory cytokines (Figure 5S–V). Furthermore, both Nile Red staining (Figure 5W,X) and analysis of lipid metabolic‐related gene expression (Figure 5Y) indicated that Eepd1 overexpression attenuated CIH‐induced lipid accumulation in hepatocytes. All the results were also observed in the absence of PAOA (Figure S4F–V, Supporting Information). Collectively, our findings suggest that the CIH‐mediated reduction in Eepd1 expression exacerbates the progression of NASH.

### 2.5 Hepatocyte‐Specific Eepd1 Deficiency Exacerbates CIH‐Induced NASH Occurrence

**Figure 6:**
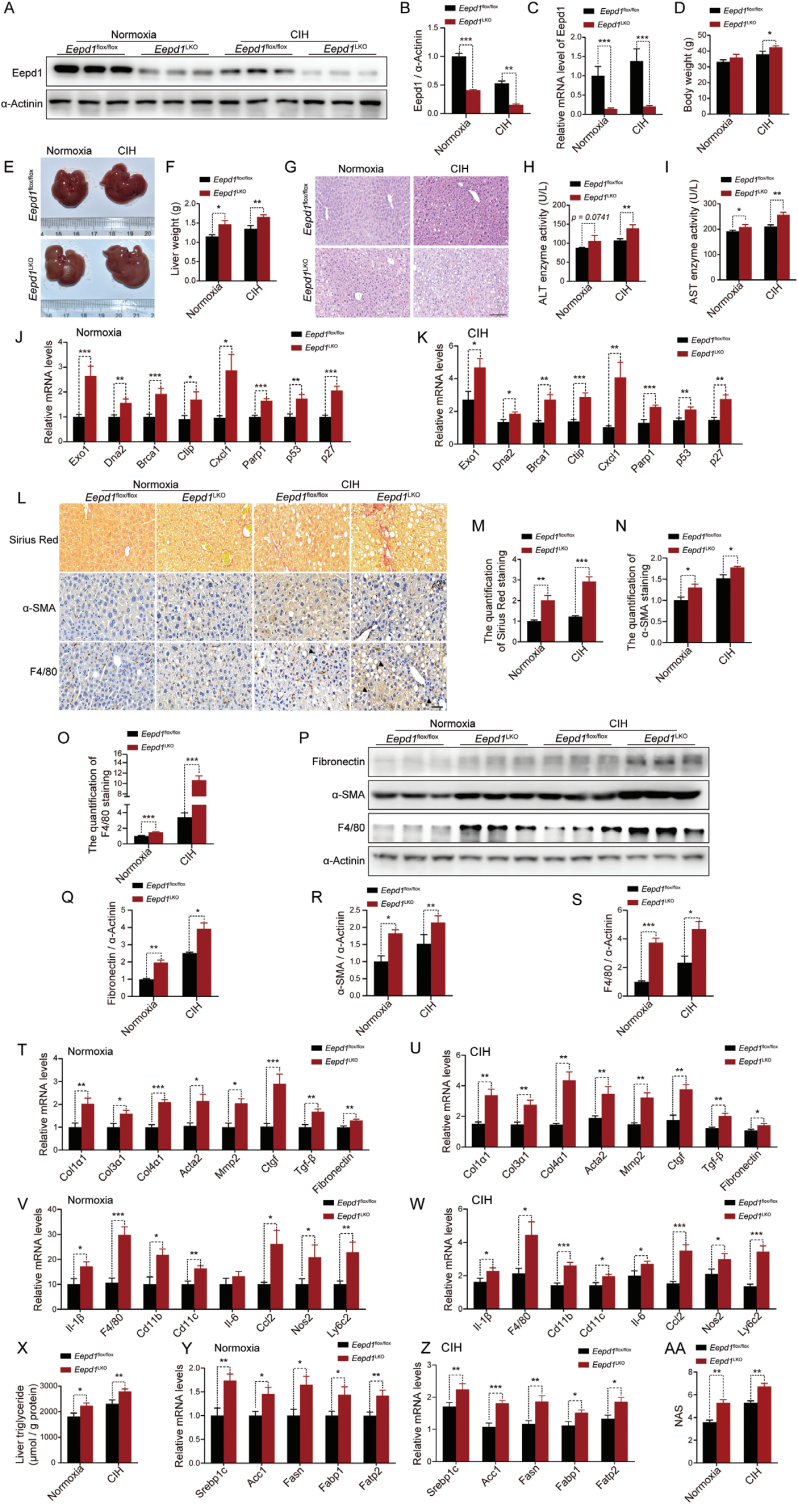
Hepatocyte‐specific Eepd1 deficiency exacerbates CIH‐induced NASH. *Eepd1* ^LKO^ and *Eepd1* ^flox/flox^ mice (8 weeks old) were fed a high‐fat diet for 4 weeks and exposed to CIH for 10 weeks. A) The protein levels of Eepd1 in the livers of *Eepd1* ^LKO^ and *Eepd1* ^flox/flox^ mice exposed to CIH (10 weeks) or normoxia (*n* = 3 per group). B) Quantitative analysis of the Eepd1 protein levels from (A). (C) The mRNA levels of *Eepd1* in the livers of *Eepd1* ^LKO^ and *Eepd1* ^flox/flox^ mice exposed to CIH (10 weeks) or normoxia (*n* = 7). D) Body weight of *Eepd1* ^LKO^ and *Eepd1* ^flox/flox^ mice exposed to CIH (10 weeks) or normoxia (*n* = 7). E) Representative images of liver morphology from *Eepd1* ^LKO^ and *Eepd1* ^flox/flox^ mice exposed to CIH (10 weeks) or normoxia. F) Liver weight of *Eepd1* ^LKO^ and *Eepd1* ^flox/flox^ mice exposed to CIH (10 weeks) or normoxia (*n* = 7). G) H&E staining of liver sections from *Eepd1* ^LKO^ and *Eepd1* ^flox/flox^ mice exposed to CIH (10 weeks) or normoxia. Scale bar, 50 µm. H) Serum ALT concentrations of *Eepd1* ^LKO^ and *Eepd1* ^flox/flox^ mice exposed to CIH (10 weeks) or normoxia. (*n* = 7). I) Serum AST concentrations of *Eepd1* ^LKO^ and *Eepd1* ^flox/flox^ mice exposed to CIH (10 weeks) or normoxia (*n* = 7). J) The mRNA levels of DNA damage‐related genes in the livers of *Eepd1* ^LKO^ and *Eepd1* ^flox/flox^ mice exposed to normoxia (*n* = 7). K) The mRNA levels of DNA damage‐related genes in the livers of *Eepd1* ^LKO^ and *Eepd1* ^flox/flox^ mice exposed to CIH (10 weeks) (*n* = 7). L) Representative Sirius Red staining and immunohistochemical staining (α‐SMA, F4/80) of livers to *Eepd1* ^LKO^ and *Eepd1* ^flox/flox^ mice exposed to CIH (10 weeks) or normoxia. Scale bar, 50 µm. The black arrow represent a crown like structure. M) Quantitative analysis of Sirius Red staining from (L). N) Quantitative analysis of α‐SMA staining from (L). O) Quantitative analysis of F4/80 staining from (L). P) The protein levels of Fibronectin, α‐SMA, and F4/80 in the livers of *Eepd1* ^LKO^ and *Eepd1* ^flox/flox^ mice exposed to CIH (10 weeks) or normoxia (*n* = 3 per group). (Q) Quantitative analysis of the Fibronectin protein levels from (P). (R) Quantitative analysis of the α‐SMA protein levels from (P). S) Quantitative analysis of the F4/80 protein levels from (P). T) The mRNA levels of genes involved in fibrosis in the livers of *Eepd1* ^LKO^ and *Eepd1* ^flox/flox^ mice exposed to normoxia (*n* = 7). U) The mRNA levels of genes involved in fibrosis in the livers of *Eepd1* ^LKO^ and *Eepd1* ^flox/flox^ mice exposed to CIH (10 weeks) (*n* = 7). V) The mRNA levels of inflammatory genes in the livers of *Eepd1* ^LKO^ and *Eepd1* ^flox/flox^ mice exposed to normoxia (*n* = 7). W) The mRNA levels of inflammatory genes in the livers of *Eepd1* ^LKO^ and *Eepd1* ^flox/flox^ mice exposed to CIH (10 weeks) (*n* = 7). X) Liver triglyceride concentrations of livers to *Eepd1* ^LKO^ and *Eepd1* ^flox/flox^ mice exposed to CIH (10 weeks) or normoxia (*n* = 7). Y) The mRNA levels of lipid metabolism related genes in the livers of *Eepd1* ^LKO^ and *Eepd1* ^flox/flox^ mice exposed to normoxia (*n* = 7). Z) The mRNA levels of lipid metabolism related genes in the livers of *Eepd1* ^LKO^ and *Eepd1* ^flox/flox^ mice exposed to CIH (10 weeks). (AA) NAFLD activity score (NAS) of *Eepd1* ^LKO^ and *Eepd1* ^flox/flox^ mice exposed to CIH (10 weeks) or normoxia assessed by H&E histology. Data are presented as mean ± S.E.M. Significance was assessed by Student's *t*‐test (B, C, D, F, H, I, M, N, O, Q, R, S, T, U, X, Y, Z, AA) or Mann–Whitney *U* test (J, K, V, W). ^*^ *p *< 0.05, ^**^ *p *< 0.01, ^***^ *p *< 0.001 versus control.

To gain further insights into the role of Eepd1 in CIH‐caused NASH, we constructed mice with a liver‐specific deletion of *Eepd1* (*Eepd1* ^LKO^, **Figure** **6**A–C; Figure S5A, Supporting Information). Both *Eepd1* ^LKO^ mice and control mice were subjected to CIH or normoxia, and CIH groups were fed a parallel HFD with normoxia groups simultaneously. The *Eepd1* ^LKO^ mice displayed elevated body weight (Figure 6D; Figure S5B, Supporting Information). Moreover, *Eepd1* ^LKO^ mice also exhibited noticeably enlarged livers, accompanied by increased liver weight when compared to the control group (Figure 6E,F; Figure S5C, Supporting Information). H&E staining (Figure 6G), serum ALT (Figure 6H), and AST (Figure 6I) concentrations all revealed a more severe degree of liver injury in *Eepd1* ^LKO^ mice. Consistent with these observations, the heightened expression levels of genes related to DNA damage (Figure 6J,K) pointed to a more significant occurrence of DNA damage in the livers of *Eepd1* ^LKO^ mice. Notably, Sirius Red staining (Figure 6L,M) demonstrated that *Eepd1* ^LKO^ mice had a significantly higher level of hepatic fibrosis than control mice. IHC and immunoblot analysis consistently confirmed an increase in the protein levels of α‐SMA and fibronectin in the liver of *Eepd1* ^LKO^ mice, as compared to the control mice (Figure 6L,N,P–R). In line with this, the expression levels of genes involved in fibrogenesis were all increased in the liver of *Eepd1* ^LKO^ mice (Figure 6T,U). Furthermore, IHC (Figure 6L,O) and immunoblot analysis (Figure 6P,S) both indicated that *Eepd1* ^LKO^ mice had a more severe inflammatory response than control mice under CIH conditions. Consistently, the mRNA levels of pro‐inflammatory cytokines were also higher in *Eepd1* ^LKO^ mice than those of control mice (Figure 6V,W).

Furthermore, hepatic TG analysis (Figure 6X) and expression of genes involved in fatty acid uptake and synthesis (Figure 6Y,Z) all revealed a significant increase in lipid accumulation in the liver of *Eepd1* ^LKO^ mice. Moreover, the expression levels of genes related to fatty acid oxidation were downregulated in the livers of *Eepd1* ^LKO^ mice, under both normoxia and CIH conditions (Figure S5D,E, Supporting Information). Additionally, we observed a minimal downregulation of genes involved in lipolysis (Figure S5F,G, Supporting Information).

In line with this observation, NAS determined from the assessment of the H&E staining was elevated in the livers of *Eepd1* ^LKO^ mice compared to the control mice (Figure 6AA; Figure S5H, Supporting Information).

### 2.6 CIH‐Caused Autophagy Leads to the Degradation of Eepd1

**Figure 7:**
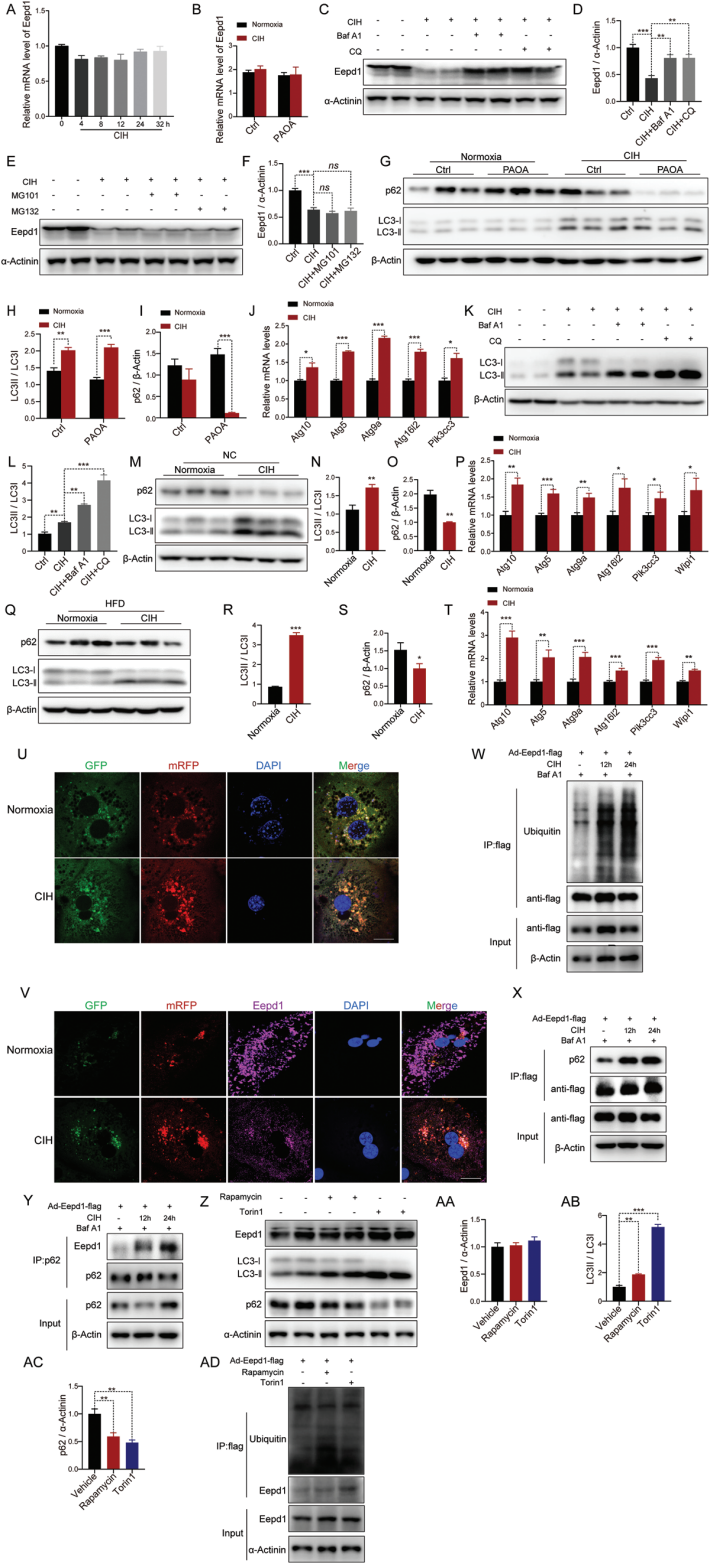
Eepd1 is degraded via autophagy under CIH. A) The mRNA levels of *Eepd1* in the primary hepatocytes subject to CIH. B) The mRNA levels of *Eepd1* in the primary hepatocytes treated with PAOA (450 µm, 32 h) under CIH (32 h) or normoxia condition. C) The protein levels of Eepd1 in the primary hepatocytes treated with Baf A1 (100 nm, 4 h) or CQ (10 µm, 12 h) under CIH (32 h) or normoxia condition (*n* = 2 per group). D) Quantitative analysis of the Eepd1 protein levels was conducted based on four experimental replicates from the two independent Western Blot results presented in panels (C) and Figure S6A (Supporting Information). E) The protein levels of Eepd1 in the primary hepatocytes treated with MG132 (10 µm, 12 h) or MG101 (10 µm, 12 h) under CIH (32 h) or normoxia condition (*n* = 2 per group). F) Quantitative analysis of the Eepd1 protein levels was conducted based on four experimental replicates from the two independent Western Blot results presented in panels (E) and Figure S6B (Supporting Information). G) The protein levels of p62 and LC3ǀ/ǁ in the primary hepatocytes treated with PAOA (450 µm, 32 h) under CIH (32 h) or normoxia condition (*n* = 3 per group). H) Quantitative analysis of LC3ǁ/LC3ǀ ratio from (G). I) Quantitative analysis of the p62 protein levels from (G). J) The mRNA levels of autophagy‐related genes in the primary hepatocytes treated with PAOA (450 µm, 32 h) under CIH (32 h) or normoxia condition. K) The protein levels of LC3ǀ/ǁ in the primary hepatocytes treated with Baf A1 (100 nm, 4 h) or CQ (10 µm, 12 h) under CIH (32 h) or normoxia condition (*n* = 2 per group). L) Quantitative analysis of LC3ǁ/LC3ǀ ratio were conducted based on four experimental replicates from the two independent Western Blot results presented in panels (K) and Figure S6D (Supporting Information). M) The protein levels of p62 and LC3ǀ/ǁ in the livers of NC‐fed mice exposed to CIH (10 weeks) or normoxia (*n* = 3 per group). N) Quantitative analysis of LC3ǁ/LC3ǀ ratio from (M). O) Quantitative analysis of the p62 protein levels from (M). P) The mRNA levels of autophagy‐related genes in the livers of NC‐fed mice exposed to CIH (10 weeks) or normoxia. Q) The protein levels of p62 and LC3ǀ/ǁ in the livers of HFD‐fed mice exposed to CIH (10 weeks) or normoxia (*n* = 3 per group). (R) Quantitative analysis of LC3ǁ/LC3ǀ ratio from (Q). S) Quantitative analysis of the p62 protein levels from (Q). T) The mRNA levels of autophagy‐related genes in the livers of HFD‐fed mice exposed to CIH (10 weeks) or normoxia. U) Immunofluorescence of LC3 in the hepatocytes transfected with mRFP‐GFP‐LC3 plasmid under CIH (32 h) or normoxia condition. Nuclei were counterstained with DAPI. Scale bar, 20 µm. V) Immunofluorescence of LC3 and Eepd1 in the hepatocytes transfected with mRFP‐GFP‐LC3 plasmid under CIH (32 h) or normoxia condition. Nuclei were counterstained with DAPI. Scale bar, 20 µm. W) Ubiquitinated Eepd1 levels in hepatocytes infected with Ad‐Eepd1‐flag and treated with Baf A1 (100 nm, 4 h) under the CIH condition (12 h and 24 h). X) p62 levels in hepatocytes infected with Ad‐Eepd1‐flag and treated with Baf A1 (100 nm, 4 h) under the CIH condition (12 h and 24 h). Y) Eepd1 levels in hepatocytes infected with Ad‐Eepd1‐flag and treated with Baf A1 (100 nm, 4 h) under the CIH condition (12 h and 24 h). Z) The protein levels of Eepd1, p62 and LC3ǀ/ǁ in the hepatocytes treated with Rapamycin (200 nm, 4 h) or Torin1 (250 nm, 4 h) (*n* = 2 per group). AA) Quantitative analysis of the Eepd1 protein levels was conducted based on four experimental replicates from the two independent Western Blot results presented in panels (Z) and Figure S6K (Supporting Information). AB) Quantitative analysis of LC3ǁ/LC3ǀ ratio was conducted based on four experimental replicates from the two independent Western Blot results presented in panels (Z) and Figure S6K (Supporting Information). AC) Quantitative analysis of the p62 protein levels was conducted based on four experimental replicates from the two independent Western Blot results presented in panels (Z) and Figure S6K (Supporting Information). AD) Ubiquitinated Eepd1 levels in hepatocytes infected with Ad‐Eepd1‐flag and treated with Rapamycin (200 nm, 4 h) or Torin1 (250 nm, 4 h). Data are presented as mean ± S.E.M. Significance was assessed by one‐way ANOVA (A, D, F, L, AA, AB, AC), Student's *t‐*test (B, H, J, N, O, R, S, T) or Mann–Whitney *U* test (I, P). *p < 0.05, **p < 0.01, ***p < 0.001 versus control.

As demonstrated by the above results, we can conclude that the CIH‐induced downregulation of Eepd1 exacerbates the progression of NASH. It is worth noting that the mRNA levels of *Eepd1* did not exhibit any significant changes under CIH conditions (**Figure** **7**A,B), pointing toward a lack of direct influence by CIH on the transcriptional regulation of *Eepd1*. Therefore, we hypothesized that CIH could potentially affect the degradation of Eepd1 protein. To test this hypothesis, we used proteasome inhibitors MG101 and MG132, as well as autophagy inhibitors Bafilomycin A1 (Baf A1) and Chloroquine (CQ) to investigate the potential reversal of Eepd1 protein levels. Interestingly, treatment with Baf A1 and CQ effectively restored the level of Eepd1 protein reduced by CIH (Figure 7C,D; Figure S6A, Supporting Information). However, the use of MG101 and MG132 did not result in a similar reversal of Eepd1 protein levels (Figure 7E,F; Figure S6B, Supporting Information). These findings suggest that the decrease in Eepd1 protein levels induced by CIH is likely mediated through activation of the autophagy pathway.

Notably, we observed a significant increase in LC3 protein levels, coupled with a reduction in the p62 protein level, in the primary hepatocytes subjected to CIH (Figure 7G–I). It is noteworthy that primary hepatocytes are typically cultured in a low‐glucose medium to prevent the activation of lipogenesis in hepatocytes.^[^ ^22^ ^]^ The low‐glucose condition led to a slight activation of basal autophagy, as evidenced by immunoblotting (Figure 7G, lines 1 to 3). These findings suggest that exposure to CIH is associated with enhanced autophagic flux. The upregulated mRNA levels of autophagy‐related genes also indicated increased autophagy in primary hepatocytes under CIH (Figure 7J; Figure S6C, Supporting Information). Furthermore, treatment with Baf A1 and CQ effectively accumulated the level of LC3‐II which also indicates enhancement of autophagic flux (Figure 7K–L; Figure S6D, Supporting Information). Consistently, the LC3‐II levels were similarly elevated in the livers of mice exposed to CIH compared to the control mice (Figure 7M,N,Q,R). In addition, the hepatic protein levels of p62 were decreased (Figure 7M,O,Q,S), and mRNA levels of autophagy‐related genes were upregulated, in the liver of mice exposed to CIH (Figure 7P,T). Taken together, these findings indicated that CIH exposure stimulates autophagy in the liver.

To delve deeper into the impact of CIH on autophagic flux, we utilized mRFP‐GFP‐LC3 plasmid transfection in primary mouse hepatocytes to visualize and assess the dynamics of autophagic flux. Exposure to CIH increased the number of green fluorescence puncta and red puncta per cell in CIH‐exposed cells compared to controls, providing evidence for upregulated autophagic flux (Figure 7U). We subsequently examined the binding of Eepd1 to LC3 puncta by immunostaining Eepd1 in hepatocytes transfected with mRFP‐GFP‐LC3 plasmids (Figure 7V). Immunofluorescence analysis revealed that CIH exposure reduced the signals of Eepd1 (Figure 7V; Figure S6E, Supporting Information). Interestingly, only a minor fraction of Eepd1 co‐localized with LC3 under normoxia, but a significant portion of Eepd1 was found to co‐localize with LC3 under CIH conditions (Figure 7V; Figure S6E, Supporting Information). These findings indicated that Eepd1 acts as an autophagy substrate that binds to LC3.

We proceeded to investigate whether Eepd1 interacts with LC3 via tag and adaptor. Typically, the universal tag used is ubiquitin. Notably, our results revealed a considerable rise in the level of ubiquitin within Eepd1 during CIH (Figure 7W). Next, we analyzed the expression of common adaptors in hepatocytes and identified p62 as the most highly expressed (Figure S6F–I, Supporting Information). Moreover, p62 mRNA levels were observed to be increased during CIH (Figure S6J, Supporting Information). This led us to speculate that p62 might serve as the adaptor facilitating the binding of Eepd1 to LC3. To validate this, we overexpressed Eepd1 in primary hepatocytes using Ad‐Eepd1‐flag. Subsequently, we treated the hepatocytes with an autophagy inhibitor and immunoprecipitated Eepd1 from cell lysates. Our results showed that p62 was co‐immunoprecipitated with Eepd1 (Figure 7X). We also performed a reverse co‐immunoprecipitation by first pulling down p62 from the cell lysate and identifying the presence of Eepd1 in the p62 precipitate (Figure 7Y). These results indicate that p62 interacts with Eepd1 and that Eepd1 undergoes autophagic degradation upon CIH exposure.

Additionally, we found that the autophagy induced by CIH was different from Rapamycin and Torin1‐mediated autophagy. As demonstrated by the failure to degrade Eepd1 using Rapamycin and Torin1 (Figure 7Z–AC; Figure S6K, Supporting Information), there was also no significant change in the ubiquitination level of Eepd1 when treated with Rapamycin and Torin1 (Figure 7AD).

### 2.7 CIH Induces Autophagy via Hypoxia Inducible Factor‐1α (Hif1α) Activation

**Figure 8:**
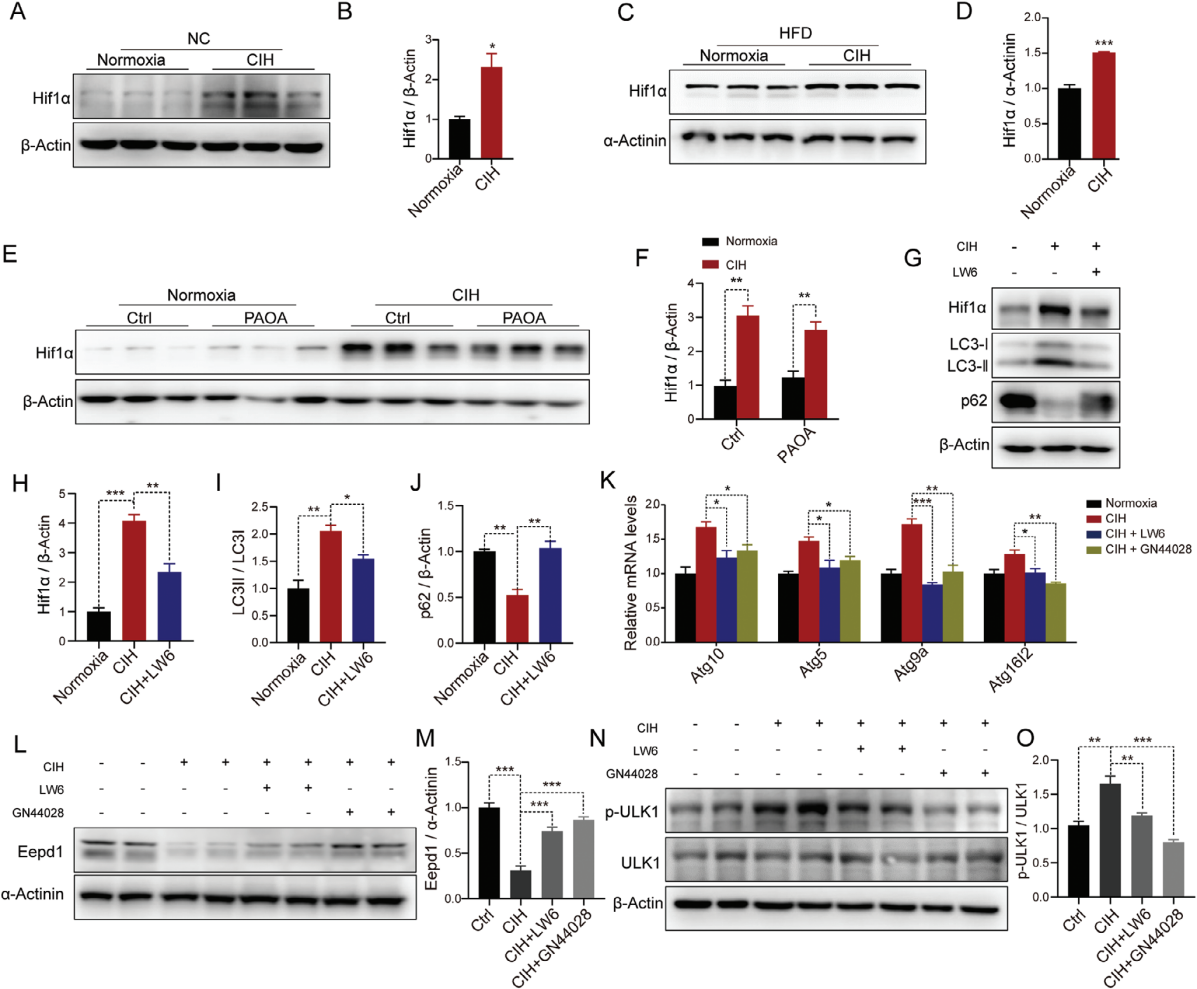
CIH promotes autophagy through Hif1α in vivo and in vitro. A) The protein levels of Hif1α in the livers of NC‐fed mice exposed to CIH (10 weeks) or normoxia (*n* = 3 per group). (B) Quantitative analysis of the Hif1α protein levels from (A). C) The protein levels of Hif1α in the livers of HFD‐fed mice exposed to CIH (10 weeks) or normoxia (*n* = 3 per group). D) Quantitative analysis of the Hif1α protein levels from (C). E) The protein levels of Hif1α in the primary hepatocytes treated with PAOA (450 µm, 32 h) under CIH (32 h) or normoxia condition (*n* = 3 per group). F) Quantitative analysis of the Hif1α protein levels from (E). G) The protein levels of Hif1α, LC3ǀ/ǁ, and p62 in the hepatocytes treated with Hif1α inhibitors LW6 (20 µm, 24 h) under CIH (32 h) condition. H) Quantitative analysis of the Hif1α protein levels performed utilizing the three independent Western Blot results presented in panels (G), Figure S7B,C (Supporting Information). I) Quantitative analysis of the LC3ǁ/LC3ǀ ratio performed utilizing the three independent Western Blot results presented in panels (G), Figure S7B,C (Supporting Information). J) Quantitative analysis of the p62 protein levels performed utilizing the three independent Western Blot results presented in panels (G), Figure S7B,C (Supporting Information). K) The mRNA levels of autophagy‐related genes in the hepatocytes treated with Hif1α inhibitors LW6 (20 µm, 24 h) or GN44028 (40 µm, 24 h) under CIH (32 h) condition. L) The protein levels of Eepd1 in the hepatocytes treated with Hif1α inhibitors LW6 (20 µm, 24 h) or GN44028 (40 µm, 24 h) under CIH (32 h) condition (*n* = 2 per group). M) Quantitative analysis of the Eepd1 protein levels was conducted based on four experimental replicates from the two independent Western Blot results presented in panels (L) and Figure S7D (Supporting Information). N) The protein levels of p‐ULK1 and ULK1 in the hepatocytes treated with Hif1α inhibitors LW6 (20 µm, 24 h) or GN44028 (40 µm, 24 h) under CIH (32 h) condition (*n* = 2 per group). O) Quantitative analysis of the p‐ULK1/ULK1 ratio was conducted based on four experimental replicates from the two independent Western Blot results presented in panels (N) and Figure S7E (Supporting Information). Data are presented as mean ± S.E.M. Significance was assessed by Student's *t‐*test (B, D, F) or one‐way ANOVA (H, I, J, K, M, O). *p < 0.05, **p < 0.01, ***p < 0.001 versus control.

Next, we attempted to elucidate the mechanism by which CIH stimulated autophagy in hepatocytes. Previous studies have demonstrated that hypoxia elevates the expression of Hif1α by generating ROS. Consistently, Dihydroethidium (DHE) staining analysis demonstrated that CIH exposure elevated ROS levels in hepatocytes (Figure S7A, Supporting Information). Notably, the expression of Hif1α was significantly up‐regulated in the livers of CIH‐exposed mice (**Figure** **8**A–D). In line with this, the protein levels of Hif1α were substantially elevated in hepatocytes under CIH condition (Figure 8E,F); by contrast, a Hif1α inhibitor (LW6) substantially blunted CIH‐caused upregulation of Hif1α (Figure 8G–H; Figure S7B,C, Supporting Information). LW6 also impaired the CIH‐induced increase in the ratio of LC3II/LC3I (Figure 8G,I; Figure S7B,C, Supporting Information) and prevented the reduction of p62 caused by CIH exposure (Figures 8G,J; Figure S7B,C, Supporting Information). Furthermore, the Hif1α inhibitor also attenuated CIH‐mediated increase in the expression of autophagy‐related genes (Figure 8K). The protein levels of Eepd1 were also restored by LW6 (Figure 8L–M; Figure S7D, Supporting Information). Another Hif1α inhibitor (GN44028) also exerted the same effect as LW6 (Figure 8L–M; Figure S7D, Supporting Information). The phosphorylation of ULK1, an important protein in the autophagy pathway, was also reduced when treated with LW6 and GN44028 (Figure 8N–O; Figure S8E, Supporting Information). We have also examined the protein expression level of HIF2α, an isoform of HIF1α, both in vivo and in vitro models to investigate its potential role in CIH‐induced autophagy. In line with previous reports,^[^ ^23^ ^]^ we observed a decrease in HIF2α protein expression under CIH conditions (Figure S7F–K, Supporting Information), which suggests that HIF2α may play minimal effects on the CIH‐mediated autophagy. Taken together, these findings indicate that CIH promotes autophagy by upregulating Hif1α, resulting in Eepd1 degradation.

### 2.8 Retigabine Dihydrochloride Ameliorated CIH‐Induced NASH Progression

**Figure 9:**
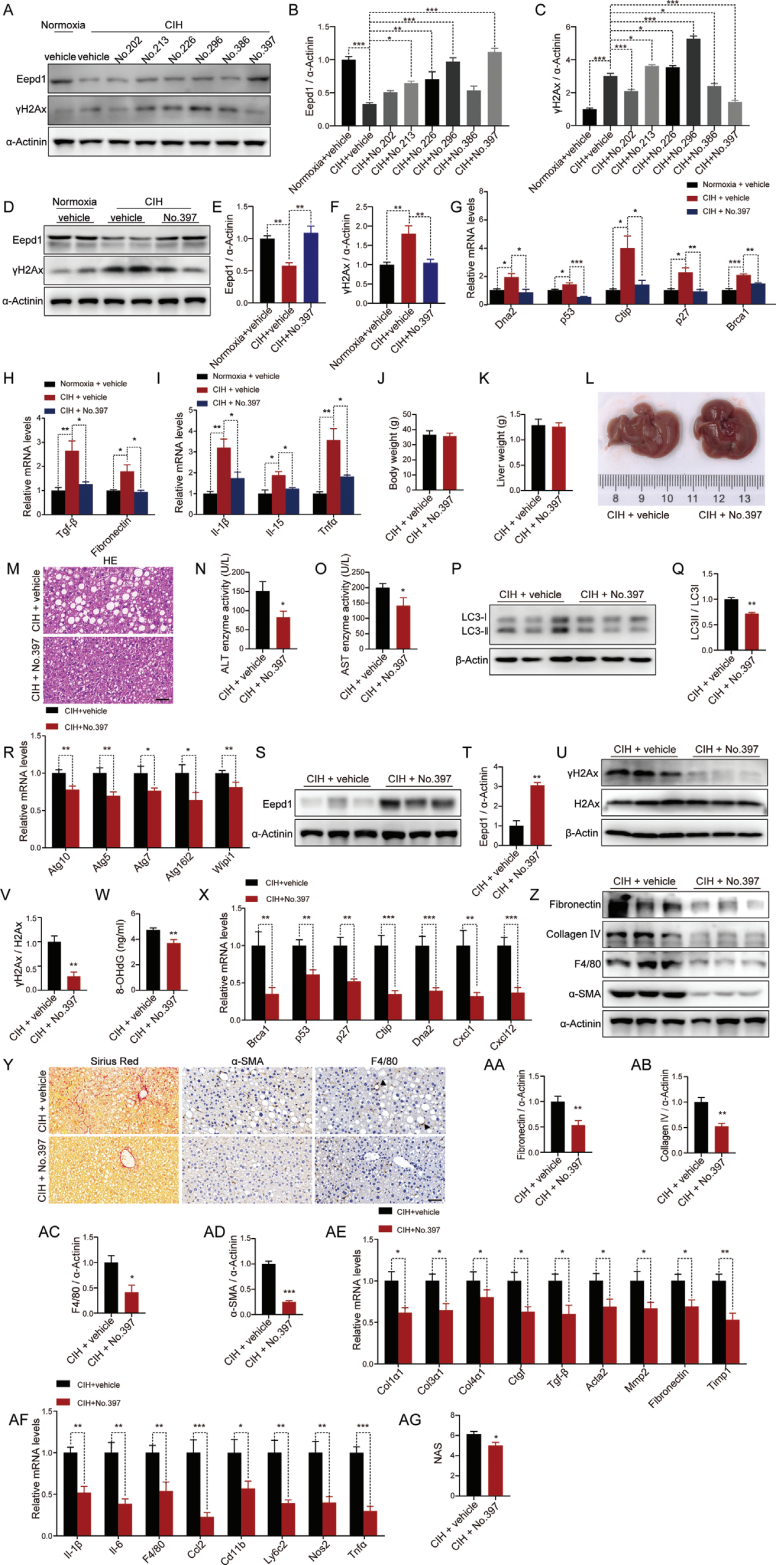
Retigabine dihydrochloride ameliorated CIH‐induced NASH progression. A) The protein levels of Eepd1 and γH2Ax in the primary hepatocytes treated with 6 drugs (10 µm, 24 h) that were selected under CIH (32 h) condition. B) Quantitative analysis of the Eepd1 protein levels performed utilizing the three independent Western Blot results presented in panels (A), Figure S8A,B (Supporting Information). C) Quantitative analysis of the γH2Ax protein levels performed utilizing the three independent Western Blot results presented in panels (A), Figure S8A,B (Supporting Information). D) The protein levels of Eepd1 and γH2Ax in the primary hepatocytes treated with Retigabine dihydrochloride (10 µm, 24 h) under CIH (32 h) condition (*n* = 2 per group). E) Quantitative analysis of the Eepd1 protein levels was conducted based on four experimental replicates from the two independent Western Blot results presented in panels (D) and Figure S8C (Supporting Information). F) Quantitative analysis of the γH2Ax protein levels was conducted based on four experimental replicates from the two independent Western Blot results presented in panels (D) and Figure S8C (Supporting Information). G) The mRNA levels of DNA damage‐related genes in the primary hepatocytes treated with Retigabine dihydrochloride (10 µm, 24 h) under CIH (32 h) condition. H) The mRNA levels of genes involved in fibrosis in the primary hepatocytes treated with Retigabine dihydrochloride (10 µm, 24 h) under CIH (32 h) condition. I) The mRNA levels of inflammatory genes in the primary hepatocytes treated with Retigabine dihydrochloride (10 µm, 24 h) under CIH (32 h) condition. (J‐AG) After 4 weeks of being fed HFD, male mice were subjected to either CIH (10 weeks) or normoxia conditions while continuing on the HFD. The mice were administered Retigabine dihydrochloride (No. 397) by gavage once daily during the final 4 weeks of CIH. J) Body weight of CIH mice treated with No. 397 (30 mg kg^−1^, 4 weeks) (*n* = 7). K) Liver weight of CIH mice treated with No. 397 (30 mg kg^−1^, 4 weeks) (*n* = 7). L) Representative images of liver morphology from CIH mice treated with No. 397 (30 mg kg^−1^, 4 weeks). (M) H&E staining of liver sections from CIH mice treated with No. 397 (30 mg kg^−1^, 4 weeks). Scale bar, 50 µm. N) Serum ALT concentrations of CIH mice treated with No. 397 (30 mg kg^−1^, 4 weeks) (*n* = 7). O) Serum AST concentrations of CIH mice treated with No. 397 (30 mg kg^−1^, 4 weeks) (*n* = 7). P) The protein levels of LC3ǀ/ǁ in the livers of CIH mice treated with No. 397 (30 mg kg^−1^, 4 weeks) (*n* = 3 per group). (Q) Quantitative analysis of LC3ǁ/LC3ǀ ratio from (P). R) The mRNA levels of autophagy‐related genes in the livers of CIH mice treated with No. 397 (30 mg kg^−1^, 4 weeks) (*n* = 7). S) The protein levels of Eepd1 in the livers of CIH mice treated with No. 397 (30 mg kg^−1^, 4 weeks) (*n* = 3 per group). T) Quantitative analysis of the Eepd1 protein levels from (S). U) The protein levels of γH2Ax in the livers of CIH mice treated with No. 397 (30 mg kg^−1^, 4 weeks) (*n* = 3 per group). V) Quantitative analysis of γH2Ax/H2Ax ratio from (U). W) Serum 8‐OHdG concentrations of CIH mice treated with No. 397 (30 mg kg^−1^, 4 weeks) (*n* = 7). X) The mRNA levels of DNA damage‐related genes in the livers of CIH mice treated with No. 397 (30 mg kg^−1^, 4 weeks) (*n* = 7). Y) Representative Sirius Red staining and immunohistochemical staining (α‐SMA, F4/80) of the livers from CIH mice treated with No. 397 (30 mg kg^−1^, 4 weeks). Scale bar, 50 µm. The black arrow represent a crown‐like structure. (Z) The protein levels of Collagen IV, Fibronectin, α‐SMA, and F4/80 in the livers of CIH mice treated with No. 397 (30 mg kg^−1^, 4 weeks) (*n* = 3 per group). (AA) Quantitative analysis of the Fibronectin protein levels from (Z). AB) Quantitative analysis of the Collagen IV protein levels from (Z). AC) Quantitative analysis of the F4/80 protein levels from (Z). AD) Quantitative analysis of the α‐SMA protein levels from (Z). AE) The mRNA levels of genes involved in fibrosis in the livers of CIH mice treated with No. 397 (30 mg kg^−1^, 4 weeks) (*n* = 7). AF) The mRNA levels of inflammatory genes in the livers of CIH mice treated with No. 397 (30 mg kg^−1^, 4 weeks) (*n* = 7). AG) NAFLD activity score (NAS) of CIH mice treated with No. 397 (30 mg kg^−1^, 4 weeks). Data are presented as mean ± S.E.M. Significance was assessed by one‐way ANOVA (B, C, E, F, G, H), Kruskal–Wallis test (I), Student's *t*‐test (J, K, O, Q, R, T, V, W, AA, AB, AC, AD, AE, AF, AG) or Mann–Whitney *U* test (N, X). ^*^ *p *< 0.05, ^**^ *p *< 0.01, ^***^ *p *< 0.001 versus control.

Given that CIH‐induced autophagy led to the degradation of Eepd1, thereby accelerating NASH progression, we sought to identify small compounds that would restore Eepd1 protein expression. We hypothesized that restoring Eepd1 protein levels using these compounds could ameliorate CIH‐induced NASH progression. To this end, we carefully selected 415 small molecule compounds for further screening, after excluding those with anti‐tumor, lethal, or inadequate safety profiles from the FDA‐Approved Drug Library. We also developed a high‐content screening system with hepatocytes overexpressing GFP‐Eepd1. Through the high‐content screening, we identified six compounds as potential hits (Table S3, Supporting Information). Subsequently, we conducted immunoblot analyses to reassess the impact of these compounds on the protein levels of Eepd1 (**Figure** **9**A–C; Figure S8A,B, Supporting Information). Among them, Retigabine dihydrochloride (designated as No.397 in Figure 9A; and Table S3, Supporting Information) emerged as the optimal compound, demonstrating significant efficacy in restoring Eepd1 protein expression (Figure 9D,E; Figure S8C, Supporting Information). Notably, the down‐regulation of γH2Ax protein expression (Figure 9D,F; Figure S8C, Supporting Information) and mRNA levels of DNA damage‐related genes in No.397‐treated hepatocytes (Figure 9G) suggested that Retigabine dihydrochloride could alleviate DNA damage induced by CIH. Additionally, No.397 substantially decreased the mRNA levels of genes associated with inflammation and fibrosis (Figure 9H,I).

To gain further insight into the impact of compound No.397 on CIH‐induced NASH, we randomly divided the CIH mice into two groups and administered compound No.397 or vehicle respectively. We observed that compound No.397 exerted a minimal impact on the body and liver weight of the CIH mice (Figure 9J–L; Figure S8D,E, Supporting Information). Remarkably, compound No.397 effectively alleviated the liver damage, as demonstrated by the H&E staining (Figure 9M) and analysis of serum ALT and AST (Figure 9N,O). Mechanistically, we found that compound No.397 suppressed CIH‐induced selective autophagy, as evidenced by reduced LC3II to LC3I ratios (Figure 9P,Q) and downregulated autophagy gene expression (Figure 9R), ultimately leading to the restoration of Eepd1 protein levels (Figure 9S,T).

In line with this, reduced expression of γH2Ax (Figure 9U,V), decreased serum 8‐OHdG levels (Figure 9W) and downregulation of genes related to DNA damage‐related genes (Figure 9X) collectively indicated that the compound No.397 mitigated DNA damage under CIH condition. Furthermore, compound No.397 exhibited robust anti‐fibrotic and anti‐inflammatory properties, as evidenced by Sirius Red staining (Figure 9Y; Figure S8F, Supporting Information), IHC staining (Figure 9Y; Figure S8G,H, Supporting Information), immunoblotting analysis (Figure 9Z–AD) and qRT‐PCR analysis (Figure 9AE,AF). Additionally, the NAS evaluation based on H&E staining also indicated that compound No.397 ameliorated the severity of NASH caused by CIH exposure (Figure 9AG; Figure S8J, Supporting Information). Taken together, our findings indicate that compound No.397 alleviated the progression of NASH under CIH conditions by reversing selective autophagy‐mediated Eepd1 degradation.

## 3 Discussion

Previous investigations have documented that intermittent hypoxia and continuous hypoxia elicit diminished appetite, reduced food consumption, and decreased body weight in murine models.^[^ ^6^, ^12^, ^21^ ^]^ However, clinical studies have revealed a contrasting scenario in OSAS patients, who tend to be obese and have an escalated appetite.^[^ ^24^ ^]^ These disparate observations between mice and humans engender a bias when exploring the impact of CIH on NASH using murine models. However, the precise underlying causes for the contrasting appetite responses in humans and mice induced by CIH remain incompletely elucidated.

To avoid disparate food intake on liver metabolism, we devised a pair‐feeding experiment with mice under CIH conditions. The mice were subjected to CIH exposure and pair‐feeding for a 10‐week duration, providing sufficient time to evaluate the direct and chronic effects of OSAS on hepatic function. Our findings demonstrate that, despite consuming the same amount of food as their normal counterparts, CIH‐exposed mice exhibit elevated body weight, intensified hepatic inflammation, and pronounced fibrosis. These observations closely resemble the phenotypic characteristics observed in OSAS patients.^[^ ^6^, ^11^, ^25^ ^]^

Numerous investigations have extensively documented that CIH can lead to ROS production, and that accumulated ROS can precipitate DNA damage.^[^ ^26^ ^]^ Consistently, we detected elevated γH2Ax levels in CIH‐exposed hepatocytes, indicating substantial DNA damage caused by CIH. Notably, the escalation of ROS induced by CIH can be primarily attributed to oxidative stress, mitochondrial dysfunction, and diminished antioxidant defenses.^[^ ^27^ ^]^ Although the occurrence of DNA damage during NASH development is well understood, the intricate modulation of DNA damage repair enzymes during the development of OSAS remains inadequately explored. Through the examination of GWAS data, noteworthy correlations have been unveiled between the SNPs situated within the DNA repair enzyme *EEPD1*, and changes in metabolic traits (including NAFLD and hyperlipidemia) as well as hypoxia‐related traits (including reticulocyte count and CT70), all of which are considered as consequence of OSAS. Our experimental findings demonstrated that CIH‐induced reductions in Eepd1 protein stability significantly exacerbated DNA damage, consequently exacerbating the progression of NASH. Moreover, both systemic and liver‐specific knockout of *Eepd1* intensified liver DNA damage, inflammation, fibrosis, and lipid accumulation under CIH conditions. In contrast, Eepd1 overexpression ameliorated DNA damage and other NASH syndromes. Collectively, these observations underscore the pivotal contribution of CIH‐mediated suppression of Eepd1 in NASH development. Notably, our investigation demonstrates that CIH induced Eepd1 degradation specifically via autophagic pathway rather than the proteasome pathway.

Autophagy, as a process of degrading intracellular components, may hold positive or negative effects on disease and health.^[^ ^28^ ^]^ Generally, autophagy has been demonstrated to be advantageous, as it can selectively target dysfunctional organelles, intracellular microorganisms, and pathogenic proteins to maintain organismal homeostasis by degrading these harmful substances.^[^ ^29^ ^]^ However, in certain contexts such as ischemic heart disease and hepatocellular cancer, augmented autophagy accelerates disease progression, underscoring its detrimental aspect.^[^ ^30^ ^]^ In this study, we provide compelling evidence supporting the notion that CIH‐triggered abnormal selective autophagy promotes the degradation of Eepd1 and exacerbates DNA damage in hepatocytes.

Given the absence of a KEFRQ‐like motif, specifically recognized by molecular chaperones, within the amino acid sequence of Eepd1 and the marked upregulation of LC3 expression under CIH conditions, we propose that Eepd1 is not subject to degradation via chaperone‐mediated autophagy or microautophagy, but rather macroautophagy. Macroautophagy can be categorized into two main forms: nonselective macroautophagy, triggered by nutrient deprivation or inducer agents (such as Rapamycin or Torin1), and selective macroautophagy, specifically targeting distinct cargoes.^[^ ^29^ ^]^ Our investigation discerned that Rapamycin or Torin1 exerted minimal influence on Eepd1 protein levels, suggesting that nonselective macroautophagy does not participate in the CIH‐induced degradation of Eepd1.

Selective macroautophagy relies on three crucial mechanisms to regulate cargo recruitment to autophagosomes: 1) direct interaction between LC3 and cargo protein, 2) interaction between LC3 and cargo protein mediated by an adaptor molecule, and 3) modification of cargo protein with a tag like ubiquitin or galectin following LC3 interaction mediated by an adaptor molecule. Our investigation revealed the absence of the LC3‐interacting region (LIR, W/F/Y1×2×3L/I/V4) motif in the amino acid sequence of Eepd1, suggesting that Eepd1 is not degraded through direct binding to LC3. We revealed that Eepd1 is ubiquitin‐tagged and interacts with LC3 through an adaptor under CIH conditions. There are six main types of conventional adapters: p62/SQSTM1, NBR1, CALCOCO2/NDP52, OPTN, TAX1BP1, and TRIMs.^[^ ^29^ ^]^ We discovered that p62/SQSTM1 is the most abundant adapter within the liver and is predominantly responsible for the autophagic degradation of Eepd1. Therefore, multiple lines of evidence demonstrate that CIH‐induced selective autophagy results in Eepd1 degradation by enhancing Eepd1 ubiquitination and promoting the interaction between p62 and Eepd1.

Collectively, our research illuminates the aggravation of NASH progression attributed to the degradation of Eepd1 induced by CIH. Furthermore, our findings suggest that interventions targeting CIH and the restoration of Eepd1 expression could serve as potential adjuncts in the therapeutic approach to NASH exacerbated by OSAS.

## 4 Experimental Section

### Animals

All animal protocols were conducted in accordance with the guidelines for the Provision and General Recommendation of Chinese Experimental Animals Administration Legislation and were approved by the Animal Care Committee of Shanghai Sixth People's Hospital affiliated to Shanghai Jiao Tong University School of Medicine (DWLL2022‐0599). Male C57BL/6J mice aged 6–8 weeks were purchased from the GemPharmatech Company (Nanjing, China). The mice were housed in a controlled environment with a temperature of 25 ± 2 °C and a humidity of 50 ± 10% under a 12‐h light/dark cycle. The *Eepd1* ^flox/flox^ mice were generated from wild‐type C57BL/6J mice using the CRISPR‐Cas9 system. For the *Eepd1* ^flox/flox^ mice, single guide RNAs (sgRNAs) were employed to target specific regions of the *Eepd1* gene. Then, the sgRNAs, Cas9 mRNA, and a donor were co‐injected into the zygotes. The 1st exon of *Eepd1* was flanked with the loxP sequence. The *Eepd1* ^flox/flox^ mice were crossbred with Albumin‐Cre mice (Jackson Laboratory, No.003574) to generate *Eepd1* hepatocyte‐specific knockout mice (*Eepd1* ^LKO^), while their littermates *Eepd1* ^flox/flox^ mice were employed as control subjects.

### CIH Model In Vivo

Male mice were fed a high‐fat diet consisting of 60% kcal fat (ResearchDiet, Cat#D12492, USA) for 4 weeks. Afterward, they were subjected to CIH in identical chambers (Oxycycler model A84; BioSpherix, Redfield, NY, USA). These chambers were equipped with programmable solenoids and flow regulators that simulated the arterial oxygen desaturation changes observed in patients with obstructive sleep apnea (OSA). The CIH was applied for 8 h d^−1^, from 9:00 am to 5:00 pm, for a total of 10 weeks. During each CIH period, the inspired oxygen fraction was gradually reduced from 21% to 5% over a period of 150 s, followed by rapid reoxygenation to room air levels within the subsequent 150 s. The normoxic control group was placed in chambers with a constant 21% O_2_, while all other experimental conditions were kept identical to those of the CIH group. Meanwhile, the drinking water with a mixture of glucose and fructose (42 g L^−1^, 55%/45%, w/w) was also supplemented during CIH.^[^ ^31^ ^]^

Regarding the in vivo experiments involving Retigabine dihydrochloride (compound No.397), the aforementioned procedure was followed to establish the CIH mouse model. Subsequently, Retigabine dihydrochloride (30 mg kg^−1^, M20428, Abmole, USA) or vehicle was administered to CIH mouse (established under CIH conditions for 6 weeks) by gavage once daily during the final 4 weeks of CIH model establishment.^[^ ^32^ ^]^

### Pair‐Feeding

In pair‐feeding experiment, the normoxia and CIH groups consumed an equal average amount of food and water per mouse every day throughout the study. According to prior studies, the food and water consumption of CIH group underwent daily monitoring, and based on the data acquired, normoxia group was administered the same quantity of food and water that CIH group had consumed on the preceding day, with this regimen being sustained on a daily basis.^[^ ^33^ ^]^

### Cell Culture

The primary hepatocytes were isolated from 8‐week‐old male C57BL/6J mice following the protocol previously described.^[^ ^34^ ^]^ After administering anesthesia, the mice were perfused with perfusate (Krebs–Ringer buffer) through the inferior vena cava, followed by perfusion with liver digestive fluid containing collagenase I (Worthington biochemical, USA). Once the perfusion was complete, the liver was harvested and digestion was terminated using ice‐cold DMEM containing 10% FBS (Gibco, USA). Then the cracked liver suspension was filtered through a 100 µm filter. The filtered cells were centrifuged at 200 g for 3 min, allowing the sediment to settle, and the supernatant was subsequently removed. The remaining precipitate was then resuspended in 45% Percoll (Pharmacia) and centrifuged again at 700 g for 10 min to eliminate dead cells. Next, the hepatocytes were resuspended with low glucose DMEM containing 10% FBS and 1% penicillin‐streptomycin (Gibco, USA) and plated on collagen (Sigma–Aldrich, USA)‐coated cell culture plates. The in vitro model of CIH was set up by exposing the hepatocytes to the CIH conditions in identical chambers. During each period of CIH, the oxygen fraction in the chamber was uniformly reduced from 21% to 1% over a 15 min period, and maintained at 1% for 10 min, then uniformly increased to 21% over a 15 min period, and maintained at 21% for 1 min. The whole process was cyclical, with a constant balance of 5% CO_2_ and nitrogen.

### ALT and AST

The blood samples were collected from the orbital vein prior to sacrificing the mice. Then the collected blood samples were centrifuged at 1,500 g for 15 min to obtain serum. Serum levels of ALT and AST were measured to evaluate liver injury according to the manufacturer's instructions (Shanghai Shensuoyoufu, China). To measure ALT activity, a mixture of lactate dehydrogenase (LDH), l‐alanine, and reduced β‐nicotinamide adenine dinucleotide (NADH) was added to the sample and incubated for 5 min. Subsequently, a mixture of l‐alanine and α‐ketoglutaric acid (α‐KG) was added and incubated for 1 min. The optical density at 340 nm was measured after both 1 min and 5 min. Finally, ALT activity was calculated according to the instructions. AST activity was determined by incubating the sample with a mixture of malate dehydrogenase (MDH), l‐aspartate, and reduced β‐nicotinamide adenine dinucleotide (NADH) for 5 min, followed by the addition of a mixture of l‐aspartate and α‐ketoglutaric acid (α‐KG) for 1 min. The optical density at 340 nm was measured after both 1 min and 5 min. AST activity was calculated according to the provided instructions.

### Triglyceride Assay

Triglyceride levels in the liver were measured with a commercially available kit (Applygene, China) according to the manufacturer's instructions. In brief, liver tissue was homogenized for lysis and lipid extraction after the addition of lysate, and the supernatant was used for protein quantification or enzymatic assays after 10 min of standing. The tissue lysate was heated and centrifuged to remove the supernatant. The optical density at 570 nm was measured after adding a mixture of lipase, glycerol kinase, glycerol phosphate oxidase, and peroxidase to the supernatant. Triglyceride content was then adjusted by the protein concentration.

### RNA Sequencing

High‐quality RNA (OD260/280:1.8‐2, OD230/260:0.4‐0.5) was extracted from the livers of mice with or without CIH (*n* = 3). RNA sequencing libraries were prepared from 4 µg of total RNA using the mRNA‐seq Library Prep Kit v2 for Illumina (Vazyme, China) according to the manufacturer's instructions. The quality of the sequencing libraries was controlled by the Agilent 2100 Bioanalyzer Instrument (Agilent Technologies, CA, USA). Libraries were sequenced in a 350 bp paired‐end run on an Illumina HiSeq PE150 Sequencing System. The 1% PhiX control library (Illumina) was spiked into each lane as the sequencing control.

### GWAS Analysis

The local GWAS analysis of OSA traits was conducted at the Sleep Center of Shanghai Jiao Tong University Affiliated Sixth People's Hospital between January 2011 and June 2019, as part of the ongoing SSHS (Shanghai Sleep Health Study) cohort. The study adhered to the principles of the Declaration of Helsinki and was registered under the number ChiCTR1900025714. It has received ethical approval from the Ethics Committee of Shanghai Sixth People's Hospital (Approval No.2021‐KY‐76), and the collection and preservation of genetic samples have been authorized by the China Human Genetic Resources Management Office (Approval No. 2022‐BC0010). Written informed consent was obtained from all participants. A total of 5773 individuals (5029 OSA patients and 744 healthy controls) were enrolled in this research. Each participant was required to complete a standardized questionnaire that collected personal information including weight, height, and medical history. The exclusion criteria encompassed the following: i) individuals under 18 years of age; ii) psychiatric disorders, chronic liver disease, or chronic kidney disease; and iii) unavailability of clinical data.

For online data mining of human genetic data, the summary statistics for total cholesterol trait (GWAS Catalog Accession: GCST90239673),^[^ ^14^ ^]^ reticulocyte count trait (GWAS Catalog Accession: GCST90002385),^[^ ^18^ ^]^ NAFLD (GWAS Catalog Accession: GCST90054782),^[^ ^16^ ^]^ and Liver Fat (GWAS Catalog Accession: GCST90016673)^[^ ^17^ ^]^ obtained from the GWAS database of EMBL's European Bioinformatics Institute (ftp.ebi.ac.uk) and sent for GWAS analysis and plotting using online version of locuszoom.js (LocusZoom.org). The p‐value criteria for genome‐wide significance was 5E‐8.

### Histological Analysis

Liver tissues harvested from mice were fixed in 4% paraformaldehyde, dehydrated, embedded in paraffin, and sliced into 5 µm thick sections on specialized glass slides for subsequent experiments. Paraffin sections were deparaffinized with xylene and rehydrated in graded alcohols to water before subsequent staining. For H&E staining, the sections were placed in hematoxylin for nuclear staining and then in eosin for cytoplasmic staining. For Sirius Red staining, the sections were placed in a staining agent for collagen coloring. The stained sections were dehydrated and transparent in gradient alcohol and xylene, sealed with neutral gum, and used for subsequent microscope photography.

### Immunohistochemistry (IHC)

The liver sections of mice were subjected to deparaffinization and rehydration, and underwent antigen repair. Then, the residual hydrogen peroxide in the sections was eliminated using 3% H_2_O_2_ in order to minimize background noise. Next, the sections were blocked with 5% bovine serum albumin (BSA) before being incubated with primary antibodies (α‐SMA, Proteintech, China, 1:200 dilution; F4/80, Proteintech, China, 1:200 dilution) overnight at 4 °C. On the following day, the secondary antibodies conjugated with horseradish peroxidase (HRP) were incubated after rewarming at 37 °C. The sections were visualized with DAB (ZLI‐9018; ZSGB‐Bio, China) and counterstained with hematoxylin for nuclei. After sealing with neutral gum, the section images were captured using a light microscope. The relative intensities of the images were quantified using Image‐Pro Plus 6.0 software.

### Immunofluorescence (IF)

For liver tissues, the paraffin sections were dewaxed to water for antigen repair and then blocked with BSA. Next, the sections were incubated with a primary antibody (γH2Ax, Cell Signaling Technology, USA, 1:200 dilution) overnight at 4 °C, and incubated with a fluorophore‐conjugated secondary antibody the next day. Nuclei were stained with DAPI (4′,6‐diamidino‐2‐phenylindole, 300 nm). Finally, the sections were sealed with an anti‐fluorescence quenching agent and imaged under a microscope. The relative intensities of the images were quantified using ImageJ software. For hepatocytes, the primary hepatocytes were fixed with 4% paraformaldehyde. After perforating with 0.5% Triton, the hepatocytes were blocked with 5% BSA and then incubated with anti‐Eepd1 (Abcam, UK, 1:200 dilution) overnight at 4 °C. After washing with PBS, the hepatocytes were incubated with Alexa 647 conjugated secondary antibodies (1:200 dilution) for 1 h. The nuclei were stained with DAPI (4′,6‐diamidino‐2‐phenylindole, 300 nm). Then, the cells were mounted and imaged by a confocal laser scanning microscope (ZEISS, Germany).

### Oil Red O staining

The primary hepatocytes were washed twice with PBS and subsequently fixed in 4% paraformaldehyde for 15 min, followed by two additional washes with PBS. Intracellular lipid deposition was assessed through staining with a working solution of 0.3% Oil Red O (Sigma), prepared by dissolving 0.3 g of Oil Red powder in 100 mL of isopropanol and filtering it, for a duration of 1 h. After rinsing with PBS, the morphology of the hepatocytes was observed using a light microscope. Image‐Pro Plus 6.0 software was used to quantitatively analyze multiple images to obtain the proportion of lipid droplets per unit area, and then statistical analysis was performed.

### Nile Red Staining

The primary hepatocytes were washed twice with PBS and subsequently fixed in 4% paraformaldehyde for 15 min, followed by two additional washes with PBS. Then 1 µm Nile Red (Sigma) was added and incubated at room temperature in the dark for 30 min. Incubation was accompanied by staining of nuclei with DAPI (4′,6‐diamidino‐2‐phenylindole, 300 nm). After completion of incubation, the cells were washed three times with PBS. The primary hepatocytes were used for subsequent microscope photography. Image J software was used to quantitatively analyze multiple images to obtain the proportion of lipid droplets per unit area, and then statistical analysis was performed.

### Dihydroethidium (DHE) Staining

DHE staining was used to assess the level of oxidative stress in primary hepatocytes. Primary hepatocytes were first washed twice with serum‐free DMEM medium and then incubated with 20 µm DHE (Beyotime) in a dark incubator at 37 °C for 30 min. The primary hepatocytes were then washed with serum‐free DMEM and immediately used for photography.

### Plasmids Transfection

GFP‐LC3 and mRFP‐GFP‐LC3 plasmids were transfected into primary hepatocytes using Lipofectamine 3000 (ThermoFisher, USA) following the manufacturer's instruction. In brief, the Lipofectamine 3000 reagent was diluted in Opti‐MEM medium (Gibco), and a master mix of plasmids was prepared by diluting them in Opti‐MEM medium and adding P3000 reagent. The diluted plasmids were then added to the diluted Lipofectamine 3000 reagent in a 1:1 ratio and incubated for 10–15 min. After incubation, the mixture was added to the primary hepatocytes. After transfection for 24 h, the cells were used for subsequent processing.

### Adenovirus Production and Transfection

The recombinant adenovirus plasmids were digested with PacI restriction enzyme (ThermoFisher, USA) and purified before transfected into 293A cells to generate adenovirus. The cesium chloride gradient centrifugation was used for the purification of recombinant adenovirus. The recombinant adenovirus was transfected into primary hepatocytes by polybrene (Sigma, USA). The medium was replaced after 12 h. The cells were used for subsequent processing.

### Immunoprecipitation (IP)

Primary hepatocytes were first infected with the adenovirus overexpressing *Eepd1* (Ad‐*Eepd1*‐flag) and then exposed to CIH. After cell treatment, the primary hepatocytes were lysed with IP lysis buffer (50 mm Tris‐HCl, 150 mm NaCl, 1 mm EDTA, 1% NP‐40, pH 7.4) supplemented with protease and phosphatase inhibitors (APExBIO, USA). The supernatant was then harvested by centrifugation. After 50 µL of each sample were left as input, the remaining supernatants were incubated with anti‐p62 antibody (CST, USA) or flag tags (Smart‐Lifesciences, China) overnight at 4 ˚C. Protein A/G magnetic beads (Thermo Fisher, USA) were added to the supernatants and incubated for 3–4 h at 4 ˚C on the next day. The conjugated beads were washed four times with wash buffer. The beads were collected after centrifugation and boiled in 2 × SDS loading buffer for 10 min, and the resulting supernatant was used for immunoblot analysis.

### Immunoblot

Total protein was isolated from liver tissue or cultured primary hepatocytes using RIPA lysis buffer (50 mm Tris‐HCl, 150 mm NaCl, 1% Triton X‐100, 0.1% SDS, 0.5% sodium deoxycholate, pH 8.0) with protease inhibitors and phosphatase inhibitors (APExBIO, USA). After separating by the SDS‐PAGE gel, the protein was transferred onto a polyvinylidene fluoride membrane (Millipore, USA). Subsequently, the membrane was blocked with 5% nonfat milk or 5% BSA for an hour, and incubated with primary antibodies (available in Table S4, Supporting Information, 1:1000 dilution) overnight at 4 ˚C. Following this, the membrane was incubated with secondary antibodies (Jackson, USA) for a further hour at room temperature. The immunoreactive bands were visualized with an enhanced chemiluminescence reagent (Millpore, USA). The ImageJ software was used to quantify the relative intensities of the bands. For all western blotting assays, a minimum of three replicates were included in each group. Statistical analysis was conducted based on at least three replicates using GraphPad Prism (version 8.0) software.

### RNA Extraction and qRT‐PCR

Total RNA was extracted from the liver tissues or primary hepatocytes with TRIzol Reagent (Thermo Fisher, USA) following the manufacturer's instructions. In brief, the primary hepatocytes were lysed with TRIzol Reagent and then layered with chloroform. The upper aqueous phase was absorbed and then the RNA was precipitated with isopropanol, washed with ethanol, and finally dissolved with ribonuclease‐free water. Then, the extracted RNA was transcribed to cDNA using the ABScript III RT Master Mix (Abclonal, China). The cDNA was used to examine the expression level of specific genes by qRT‐PCR system (Roche, Switzerland) using Universal SYBR Green Fast qPCR Mix (Abclonal, China). *18S* and *36b4* were selected as the housekeeping genes. qRT‐PCR primers are available in Table S5 (Supporting Information).

### High Content Screening

After excluding compounds exhibiting anti‐tumor activity, lethality, or inadequate safety profiles, 415 small molecule compounds were meticulously selected from the FDA‐approved drug library (TargetMol, USA, L1000) for subsequent high‐content screening. Mouse primary hepatocytes were transduced with adenovirus to overexpress GFP‐Eepd1, followed by exposure to chronic intermittent hypoxia (CIH) and treatment with the aforementioned 415 small molecule drugs. Fluorescence signals of GFP‐Eepd1 were recorded using the High Content Imaging System (ImageXpress&MetaXpress). The primary screening criterion for small molecules was the “High Content Score,” calculated based on the average fluorescence intensity per cell. Elevated High Content Scores corresponded to heightened levels of Eepd1 protein.

### Statistical Analysis

Statistical analyses were performed with GraphPad Prism (version 8.0) software. All data were presented as the mean ± standard error of the mean (S.E.M). The differences were analyzed using the Kruskal Wallis test, Mann‐Whitney *U* test, one‐way ANOVA, or Student's *t*‐test where appropriate. The normality of the data was analyzed by D'Agostino's K‐squared test, Shapiro–Wilk normality test, and Kolmogorov–Smirnov test before analysis of the significance. Student's *t*‐test or one‐way ANOVA was used for normal distribution where appropriate, and Mann–Whitney *U* test or Kruskal–Wallis test was used for non‐normal distribution. Pearson correlation analysis was used to analyze the linear correlation between the two groups of data when appropriate. Statistically significant was defined as ^*^ *p *< 0.05, ^**^ *p *< 0.01, and ^***^ *p *< 0.001.

## Conflict of Interest

The authors declare no conflict of interest.

## Author Contributions

J.L.L., S.K.Y., F.L. and S.Z.C. conceived and designed the study; J.X., Y.X., N.W., Y.Z., S.J.L., X.M.Z., X.X.L., C.C.L., Q.X.J., J.T.X., Q.Q.Q., P.H.Z. and L.M.Y. performed the experiments, J.X., J.L.L. and Y.X. analyzed data and participated in discussion of the results. J.X., J.L.L., S.Z.C., and F.L. wrote the manuscript. All authors contributed to the manuscript and approved it for publication.

## Supporting information

Supporting Information

## Data Availability

All study data are included in the article and/or supporting information.
