# Supplementary material for: Obstructive Sleep Apnea Syndrome Exacerbates NASH Progression via Selective Autophagy‐Mediated Eepd1 Degradation
Source: Adv Sci (Weinh). 2024 Jun 25;11(35):2405955. doi: 10.1002/advs.202405955 (PMC11425227; doi:10.1002/advs.202405955)
Supplement: Supplementary file 1 — Supporting Information [file ADVS-11-2405955-s001.docx]

**Supplementary information**


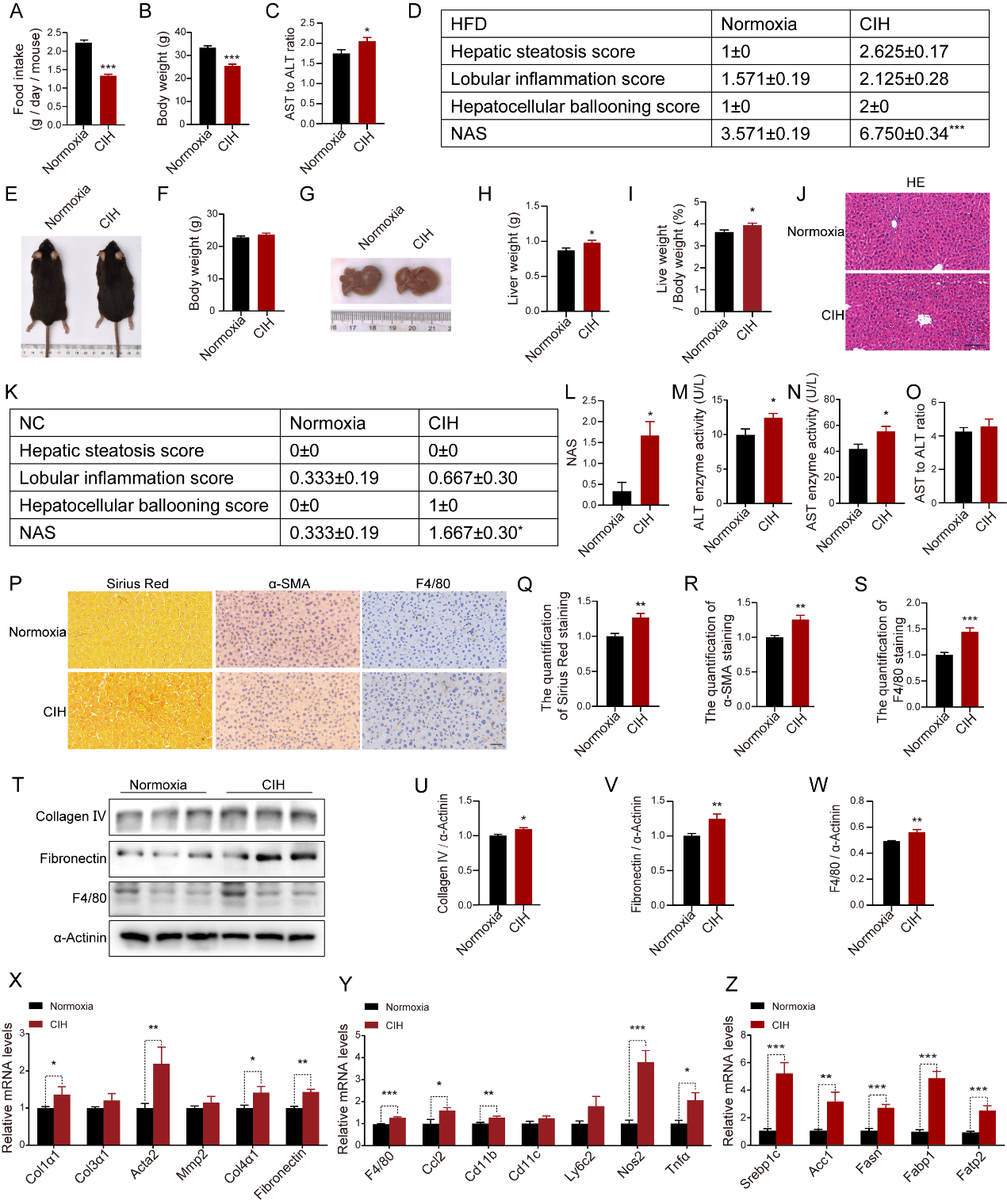


**Figure S1. CIH exacerbates NASH progression *in vivo***

**Related to Figure 1**

1. Food intake of HFD-fed mice with *ad libitum* access to the diet exposed to CIH (10 weeks) or normoxia (*n* = 7).
2. Body weight of HFD-fed mice with *ad libitum* access to the diet exposed to CIH (10 weeks) or normoxia (*n* = 7).
3. AST to ALT ratios of HFD-fed mice exposed to CIH (10 weeks) or normoxia (*n* = 10).
4. The NAFLD activity score (NAS) of HFD-fed mice exposed to CIH (10 weeks) or normoxia was calculated by summing the scores of steatosis, lobular inflammation, and hepatocellular ballooning, all of which were assessed by H&E histology.
5. Representative images of NC-fed mice exposed to CIH (10 weeks) or normoxia.
6. Body weight of NC-fed mice exposed to CIH (10 weeks) or normoxia (*n* = 7).
7. Representative images of liver morphology from NC-fed mice exposed to CIH (10 weeks) or normoxia.
8. Liver weight of NC-fed mice exposed to CIH (10 weeks) or normoxia (*n* = 7).
9. Liver weight to body weight ratios of NC-fed mice exposed to CIH (10 weeks) or normoxia (*n* = 7).
10. H&E staining of liver sections from NC-fed mice exposed to CIH (10 weeks) or normoxia. Scale bar, 50 μm.
11. The NAFLD activity score (NAS) of NC-fed mice exposed to CIH (10 weeks) or normoxia was calculated by summing the scores of steatosis, lobular inflammation, and hepatocellular ballooning, all of which were assessed by H&E histology.
12. NAFLD activity score (NAS) of NC-fed mice exposed to CIH (10 weeks) or normoxia.
13. Serum ALT concentrations of NC-fed mice exposed to CIH (10 weeks) or normoxia (*n* = 7).
14. Serum AST concentrations of NC-fed mice exposed to CIH (10 weeks) or normoxia (*n* = 7).
15. AST to ALT ratios of NC-fed mice exposed to CIH (10 weeks) or normoxia (*n* = 7).
16. Representative Sirius Red staining and immunohistochemical staining (α-SMA, F4/80) of livers to NC-fed mice exposed to CIH (10 weeks) or normoxia. Scale bar, 50 μm.
17. Quantitative analysis of Sirius Red staining from (P).
18. Quantitative analysis of α-SMA staining from (P).
19. Quantitative analysis of F4/80 staining from (P).
20. The protein levels of Collagen IV, Fibronectin and F4/80 in the livers of NC-fed mice exposed to CIH (10 weeks) or normoxia (*n* = 3 / group).
21. Quantitative analysis of the Collagen IV protein levels from (T).
22. Quantitative analysis of the Fibronectin protein levels from (T).
23. Quantitative analysis of the F4/80 protein levels from (T).
24. The mRNA levels of genes involved in fibrosis in the livers of NC-fed mice exposed to CIH (10 weeks) or normoxia.
25. The mRNA levels of inflammatory genes in the livers of NC-fed mice exposed to CIH (10 weeks) or normoxia.
26. The mRNA levels of lipid metabolism related genes in the livers of NC-fed mice exposed to CIH (10 weeks) or normoxia.

Data are presented as mean ± S.E.M. Significance was assessed by Student’s *t* test (A, B, C, D, F, H, I, K, L, M, N, Q, R, S, U, V, W, Y, Z) or Mann-Whitney *U* test (O, X). * *p*< 0.05, ** *p*< 0.01, *** *p*< 0.001 versus control.


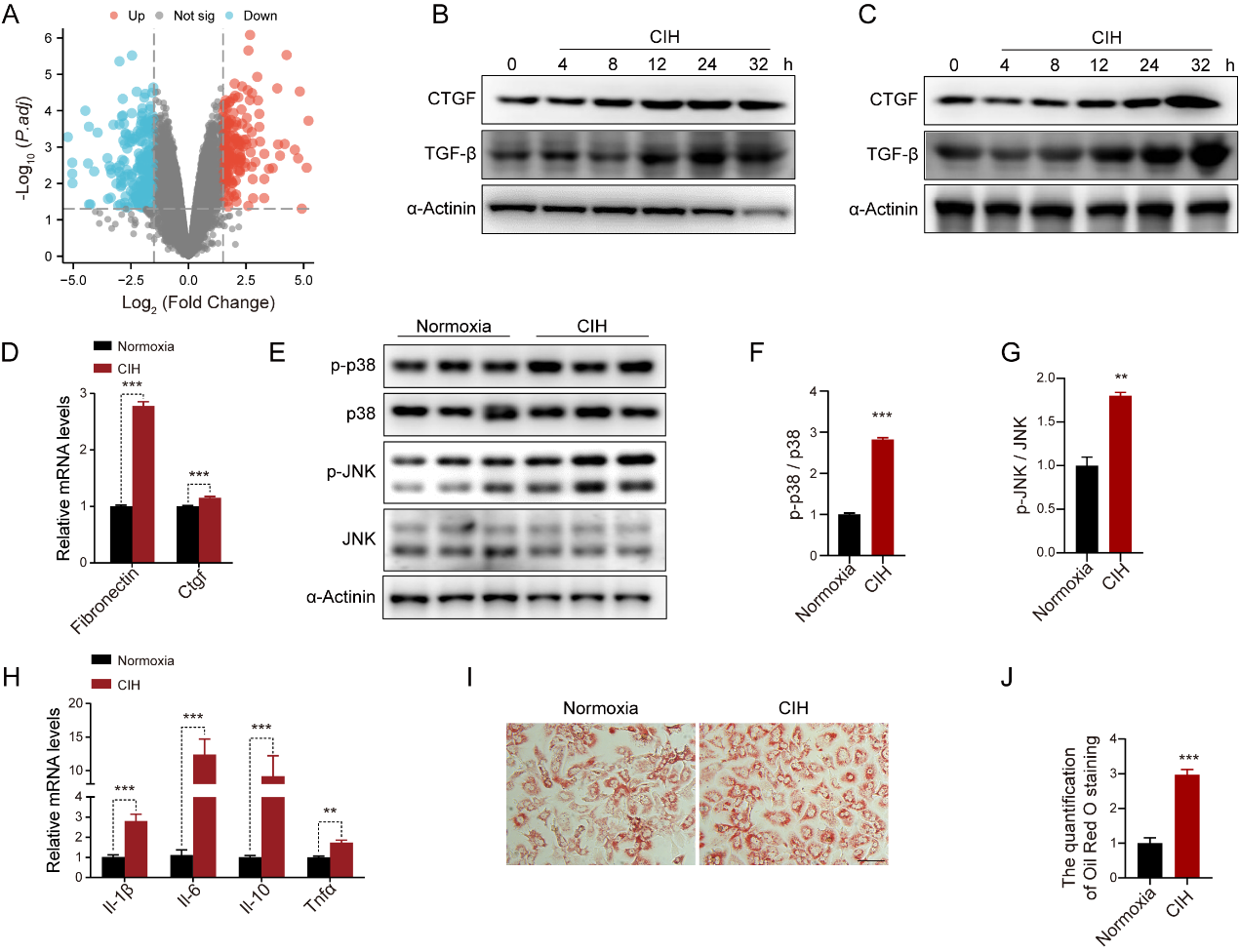


**Figure S2. CIH aggravates fibrosis, inflammation and lipid accumulation in hepatocytes without PAOA stimulation**

**Related to Figure 2**

1. The Volcano plot of RNA-seq data obtained from livers of HFD-fed mice exposed to CIH (10 weeks) or normoxia.
2. The protein levels of CTGF and TGF-β in the primary hepatocytes subjected to CIH treated with PAOA (450 μM, 32 h).
3. The protein levels of CTGF and TGF-β in the primary hepatocytes subjected to CIH treated with PAOA (450 μM, 32 h).
4. The mRNA levels of genes involved in fibrosis in the primary hepatocytes under CIH (32 h) or normoxia condition.
5. The protein levels of p-p38 and p-JNK in the primary hepatocytes under CIH (32 h) or normoxia condition (*n* = 3 / group).
6. Quantitative analysis of p-p38/p38 ratio from (E).
7. Quantitative analysis of p-JNK/JNK ratio from (E).
8. The mRNA levels of inflammatory genes in the primary hepatocytes under CIH (32 h) or normoxia condition.
9. The Oil Red O staining in the primary hepatocytes under CIH (32 h) or normoxia condition.
10. Quantitative analysis of Oil Red O staining from (I).

Data are presented as mean ± S.E.M. Significance was assessed by Mann-Whitney *U* test (H) or Student’s *t* test (D, F, G, J). * *p* < 0.05, ** *p*< 0.01, *** *p*< 0.001 versus control.


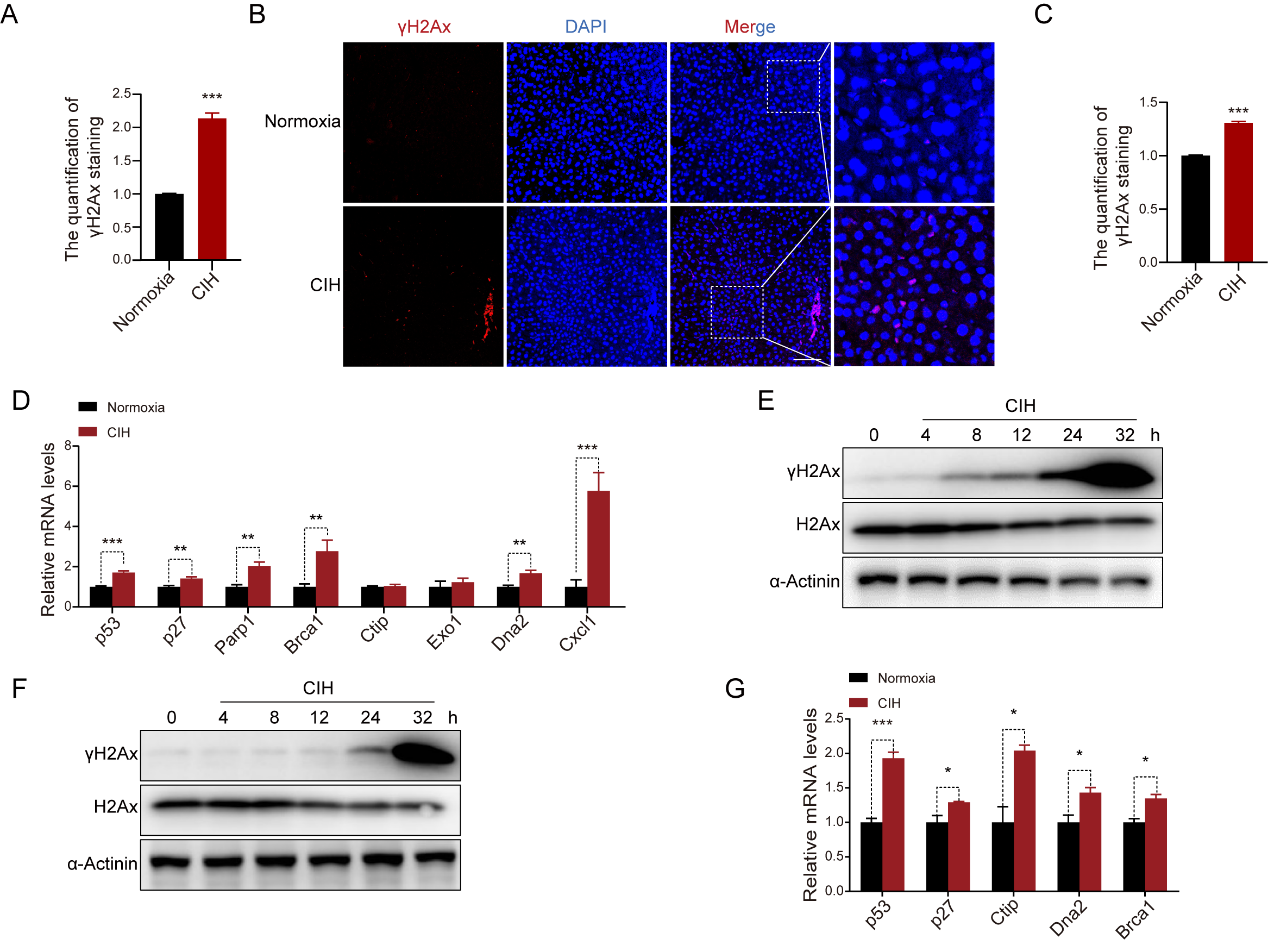


**Figure S3. CIH exacerbates DNA damage**

**Related to Figure 3**

1. Quantitative analysis of γH2Ax staining from Figure 3 (C).
2. Immunofluorescence of γH2Ax of liver sections from NC-fed mice exposed to CIH (10 weeks) or normoxia. Nuclei were counterstained with DAPI. Scale bar, 50 μm.
3. Quantitative analysis of γH2Ax staining from (B).
4. The mRNA levels of DNA damage-related genes in the livers of NC-fed mice exposed to CIH (10 weeks) or normoxia.
5. The protein levels of γH2Ax in the primary hepatocytes subjected to CIH.
6. The protein levels of γH2Ax in the primary hepatocytes subjected to CIH.
7. The mRNA levels of DNA damage-related genes in the primary hepatocytes under CIH (32 h) or normoxia condition.

Data are presented as mean ± S.E.M. Significance was assessed by Student’s *t* test (A, C, D, G). * *p* < 0.05, ** *p*< 0.01, *** *p*< 0.001 versus control.


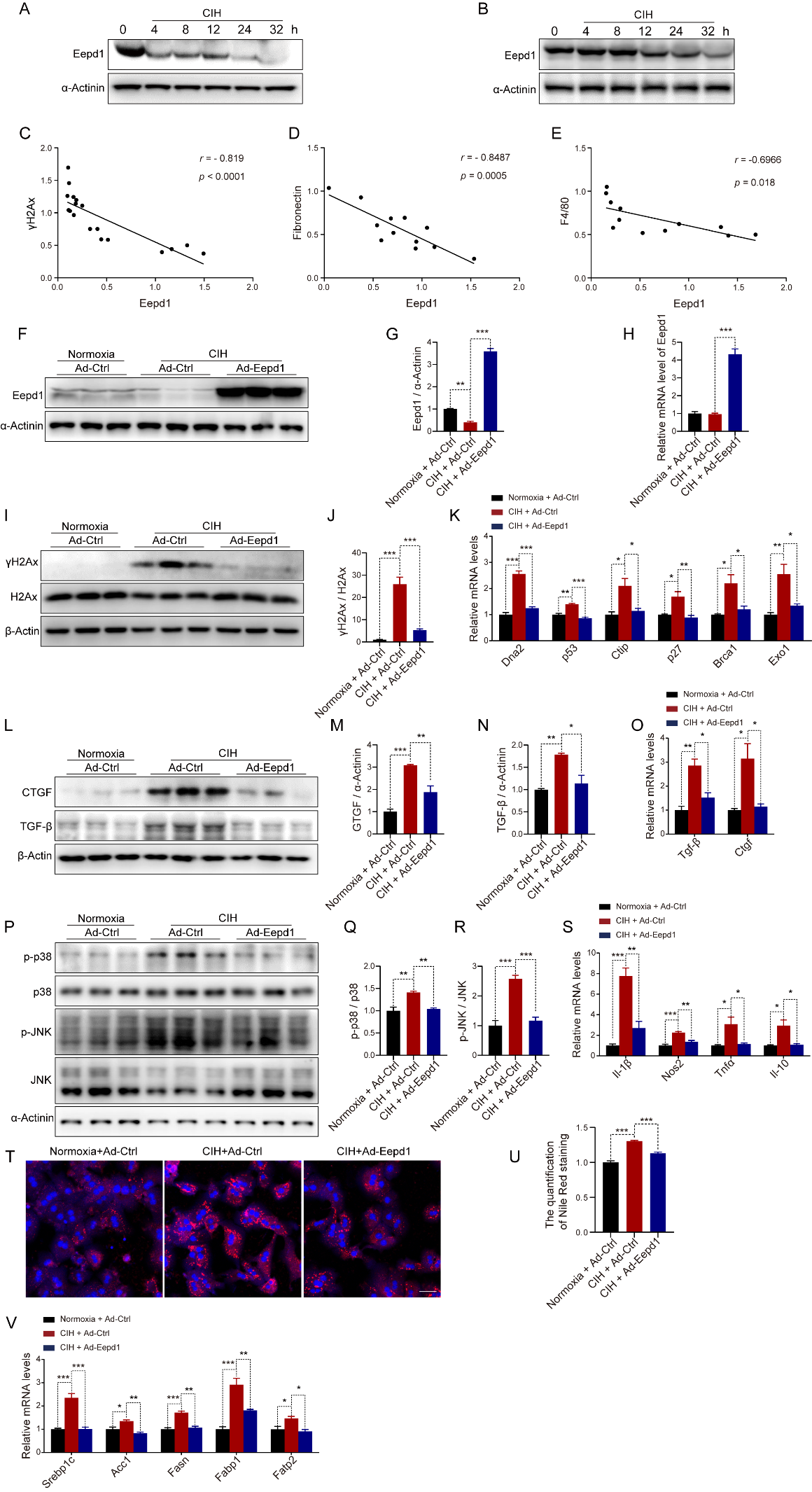


**Figure S4. Eepd1 correlates with NASH progression and overexpression of Eepd1 alleviates CIH-induced NASH**

**Related to Figure 5**

1. The protein levels of Eepd1 in the primary hepatocytes subjected to CIH.
2. The protein levels of Eepd1 in the primary hepatocytes subjected to CIH.
3. The correlation analysis between the Eepd1 protein levels and γH2Ax protein levels.
4. The correlation analysis between the Eepd1 protein levels and Fibronectin protein levels.
5. The correlation analysis between the Eepd1 protein levels and F4/80 protein levels.
6. The protein levels of Eepd1 in the hepatocytes overexpressed *Eepd1* under CIH (32 h) or normoxia condition (*n* = 3 / group).
7. Quantitative analysis of the protein levels of Eepd1 from (F).
8. The mRNA levels of *Eepd1* in the hepatocytes overexpressed *Eepd1* under CIH (32 h) or normoxia condition.
9. The protein levels of γH2Ax in the hepatocytes overexpressed *Eepd1* under CIH (32 h) or normoxia condition (*n* = 3 / group).
10. Quantitative analysis of γH2Ax/H2Ax ratio from (I).
11. The mRNA levels of DNA damage-related genes in the hepatocytes overexpressed *Eepd1* under CIH (32 h) or normoxia condition.
12. The protein levels of CTGF and TGF-β in the hepatocytes overexpressed *Eepd1* under CIH (32 h) or normoxia condition (*n* = 3 / group).
13. Quantitative analysis of the protein levels of CTGF from (L).
14. Quantitative analysis of the protein levels of TGF-β from (L).
15. The mRNA levels of genes involved in fibrosis in the hepatocytes overexpressed *Eepd1* under CIH (32 h) or normoxia condition.
16. The protein levels of p-p38 and p-JNK in the hepatocytes overexpressed *Eepd1* under CIH (32 h) or normoxia condition (*n* = 3 / group).
17. Quantitative analysis of p-p38/p38 ratio from (P).
18. Quantitative analysis of p-JNK/JNK ratio from (P).
19. The mRNA levels of inflammatory genes in the hepatocytes overexpressed *Eepd1* under CIH (32 h) or normoxia condition.
20. The Nile Red staining in the hepatocytes overexpressed *Eepd1* under CIH (32 h) or normoxia condition.
21. Quantitative analysis of Nile Red staining from (T).
22. The mRNA levels of lipid metabolism related genes in the hepatocytes overexpressed *Eepd1* under CIH (32 h) or normoxia condition.

Data are presented as mean ± S.E.M. Significance was assessed by Pearson correlation (C, D, E) or one-way ANOVA (G, H, J, K, M, N, Q, R, S, U, V). * *p* < 0.05, ***p* < 0.01, *** *p* < 0.001 versus control.


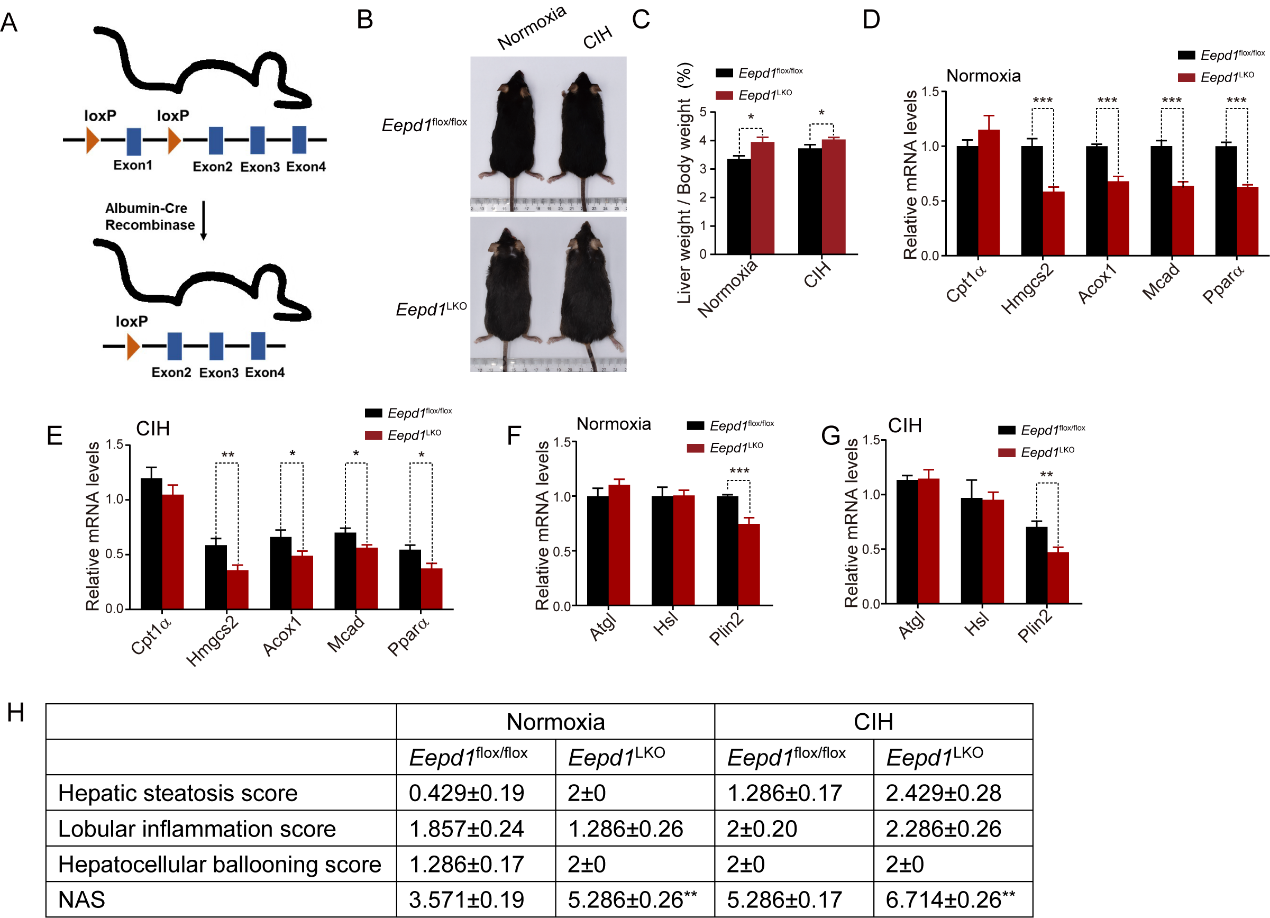


**Figure S5. Hepatocyte-specific Eepd1 deficiency exacerbates CIH-induced NASH**

**Related to Figure 6**

1. The schema of *Eepd1*^LKO^ mice construction
2. Representative images of *Eepd1*^LKO^ and *Eepd1*^flox/flox^ mice exposed to CIH (10 weeks) or normoxia.
3. Liver weight to body weight ratios of *Eepd1*^LKO^ and *Eepd1*^flox/flox^ mice exposed to CIH (10 weeks) or normoxia (*n* = 7).
4. The mRNA levels of β-oxidation related genes in the livers of *Eepd1*^LKO^ and *Eepd1*^flox/flox^ mice exposed to normoxia.
5. The mRNA levels of β-oxidation related genes in the livers of *Eepd1*^LKO^ and *Eepd1*^flox/flox^ mice exposed to CIH (10 weeks).
6. The mRNA levels of lipolysis related genes in the livers of *Eepd1*^LKO^ and *Eepd1*^flox/flox^ mice exposed to normoxia.
7. The mRNA levels of lipolysis related genes in the livers of *Eepd1*^LKO^ and *Eepd1*^flox/flox^ mice exposed to CIH (10 weeks).
8. The NAFLD activity score (NAS) of *Eepd1*^LKO^ and *Eepd1*^flox/flox^ mice exposed to CIH (10 weeks) or normoxia was calculated by summing the scores of steatosis, lobular inflammation, and hepatocellular ballooning, all of which were assessed by H&E histology.

Data are presented as mean ± S.E.M. Significance was assessed by Student’s *t* test (C, E, F, G, H) or Mann-Whitney *U* test (D). * *p*< 0.05, ** *p*< 0.01, *** *p*< 0.001 versus control.


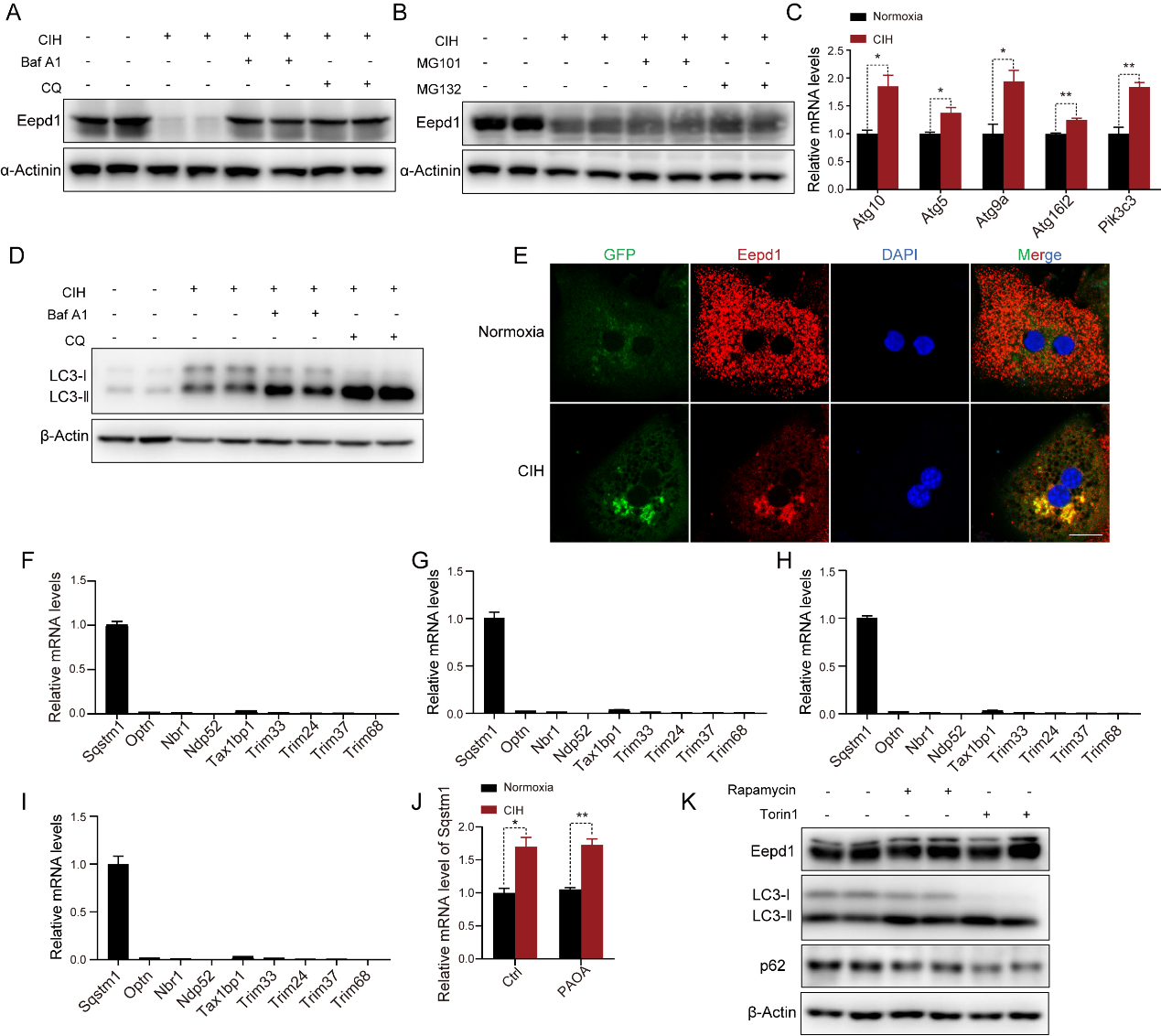


**Figure S6. Eepd1 is degraded via autophagy under CIH**

**Related to Figure 7**

1. The protein levels of Eepd1 in the primary hepatocytes treated with Baf A1 (100 nM, 4 h) or CQ (10 μM, 12 h) under CIH (32 h) or normoxia condition (n = 2 / group).
2. The protein levels of Eepd1 in the primary hepatocytes treated with MG132 (10 μM, 12 h) or MG101 (10 μM, 12 h) under CIH (32 h) or normoxia condition (*n* = 2 / group).
3. The mRNA levels of autophagy-related genes in the primary hepatocytes under CIH (32 h) or normoxia condition.
4. The protein levels of LC3ǀ/ǁ in the primary hepatocytes treated with Baf A1 (100 nM, 4 h) or CQ (10 μM, 12 h) under CIH (32 h) or normoxia condition (*n* = 2 / group).
5. Immunofluorescence of LC3 and Eepd1 in the primary hepatocytes transfected with GFP-LC3 plasmid under CIH (32 h) or normoxia condition. Nuclei were counterstained with DAPI. Scale bar, 20 μm.
6. The mRNA levels of adaptors in the primary hepatocytes treated with PAOA (450 μM, 32 h) under CIH (32 h) or normoxia condition.
7. The mRNA levels of adaptors in the primary hepatocytes under normoxia condition.
8. The mRNA levels of adaptors in the primary hepatocytes treated with PAOA (450 μM, 32 h).
9. The mRNA levels of adaptors in the primary hepatocytes under CIH (32 h) condition.
10. The mRNA levels of p62 in the primary hepatocytes treated with PAOA (450 μM, 32 h) under CIH (32 h) condition.
11. The protein levels of Eepd1, p62 and LC3ǀ/ǁ in the hepatocytes treated with Rapamycin (200 nM, 4 h) or Torin1 (250 nM, 4 h) (*n* = 2 / group).

Data are presented as mean ± S.E.M. Significance was assessed by one-way ANOVA (C, J) or Student’s *t* test (F, G, H, I). * *p*< 0.05, ** *p*< 0.01, *** *p* < 0.001 versus control.


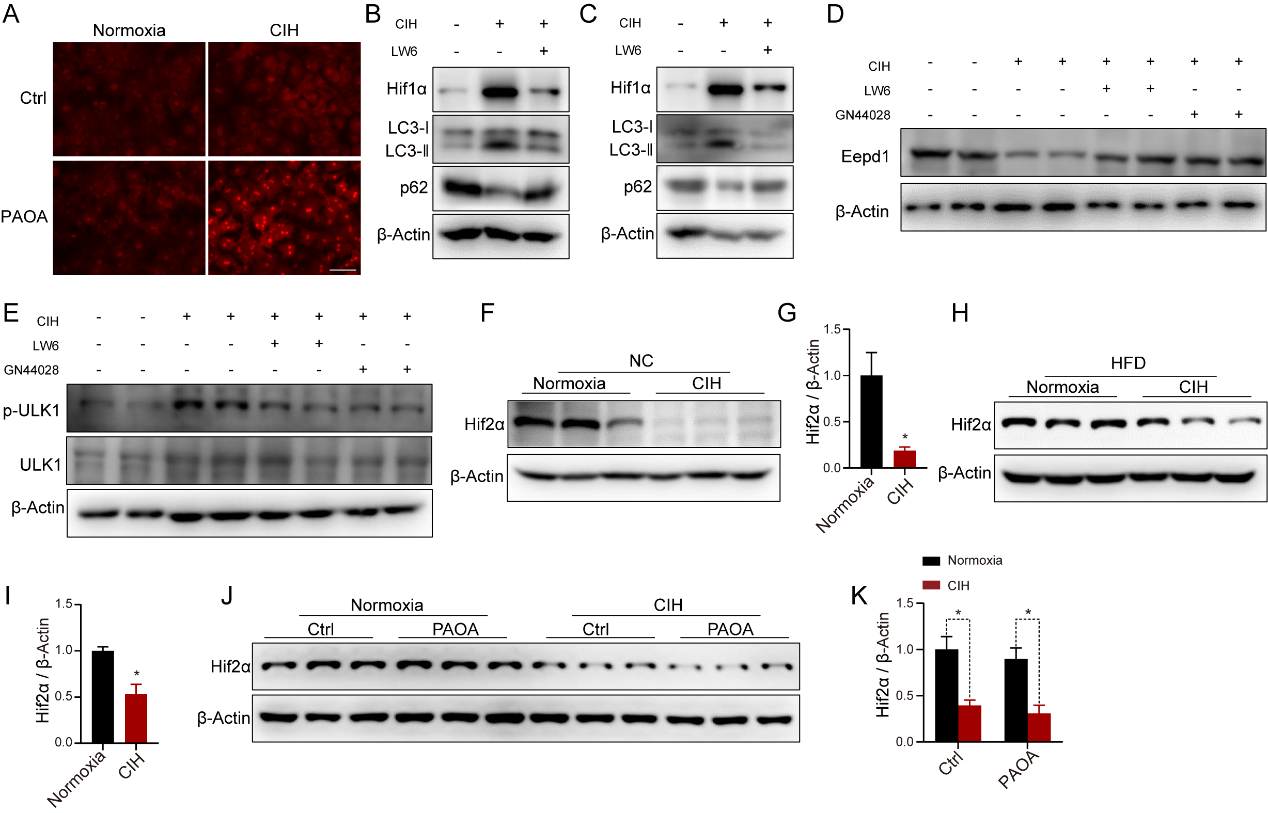


**Figure S7. CIH promotes autophagy through Hif1α *in vitro***

**Related to Figure 8**

1. The DHE staining of hepatocytes.
2. The protein levels of Hif1α, LC3ǀ/ǁ and p62 in the hepatocytes treated with Hif1α inhibitors LW6 (20 μM, 24 h) under CIH (32 h) condition.
3. The protein levels of Hif1α, LC3ǀ/ǁ and p62 in the hepatocytes treated with Hif1α inhibitors LW6 (20 μM, 24 h) under CIH (32 h) condition.
4. The protein levels of Eepd1 in the hepatocytes treated with Hif1α inhibitors LW6 (20 μM, 24 h) or GN44028 (40 μM, 24 h) under CIH (32 h) condition (*n* = 2 / group).
5. The protein levels of p-ULK1 and ULK1 in the hepatocytes treated with Hif1α inhibitors LW6 (20 μM, 24 h) or GN44028 (40 μM, 24 h) under CIH (32 h) condition (*n* = 2 / group).
6. The protein levels of Hif2α in the livers of NC-fed mice exposed to CIH (10 weeks) or normoxia (*n* = 3 / group).
7. Quantitative analysis of the Hif2α protein levels from (F).
8. The protein levels of Hif2α in the livers of HFD-fed mice exposed to CIH (10 weeks) or normoxia (*n* = 3 / group).
9. Quantitative analysis of the Hif2α protein levels from (H).
10. The protein levels of Hif2α in the primary hepatocytes treated with PAOA (450 μM, 32 h) under CIH (10 weeks) or normoxia (*n* = 3 / group).
11. Quantitative analysis of the Hif2α protein levels from (J).

Data are presented as mean ± S.E.M. Significance was assessed by Student’s *t* test (I, K) or Mann-Whitney *U* test (G). * *p*< 0.05, ** *p*< 0.01, *** *p*< 0.001 versus control.


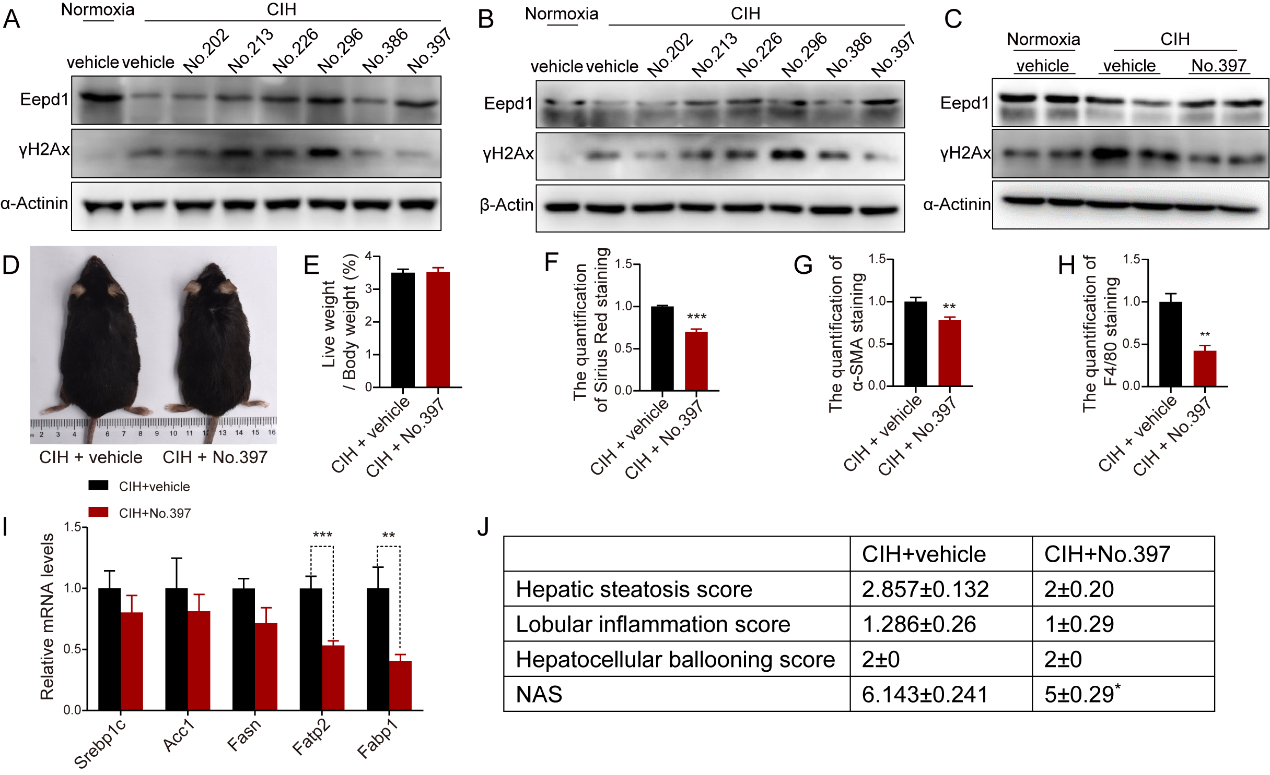


**Figure S8. Retigabine dihydrochloride ameliorated CIH-induced NASH progression**

**Related to Figure 9**

1. The protein levels of Eepd1 and γH2Ax in the primary hepatocytes treated with 6 drugs (10 μM, 24 h) that were selected under CIH (32 h) condition.
2. The protein levels of Eepd1 and γH2Ax in the primary hepatocytes treated with 6 drugs (10 μM, 24 h) that were selected under CIH (32 h) condition.
3. The protein levels of Eepd1 and γH2Ax in the primary hepatocytes treated with Retigabine dihydrochloride (10 μM, 24 h) under CIH (32 h) condition (*n* = 2 / group).
4. Representative images of CIH mice treated with No. 397 (30 mg/kg, 4 weeks).
5. Liver weight to body weight ratios of CIH mice treated with No. 397 (30 mg/kg, 4 weeks) (n = 7).
6. Quantitative analysis of Sirius Red staining from Figure 9 (Y).
7. Quantitative analysis of α-SMA staining from Figure 9 (Y).
8. Quantitative analysis of F4/80 staining from Figure 9 (Y).
9. The mRNA levels of lipid metabolism related genes in the liver of CIH mice treated with No. 397 (30 mg/kg, 4 weeks) (*n* = 7).
10. NAFLD activity score (NAS) of CIH mice with or without No. 397 as the sum of steatosis, lobular inflammation, and hepatocellular ballooning scores assessed by H&E histology.

Data are presented as mean ± S.E.M. Significance was assessed by Student’s *t* test (E, F, G, H, J) or Mann-Whitney *U* test (I). * *p*< 0.05, ** *p*< 0.01, *** *p*< 0.001 versus control.

**Table S1. The association of extant genetic polymorphisms of Eepd1 with metabolic traits are listed as follows.**

| ***EEPD1* SNPs** | **traits** | ***p*-value** | **β** | **ci** | **PubMed ID** |
| --- | --- | --- | --- | --- | --- |
| rs67050321 | LDL cholesterol levels | 6.00E-13 | 0.0202 | [0.015-0.025] | 32154731 |
|  | LDL cholesterol levels | 5.00E-20 | -0.0191502 | [0.015-0.023] | 32493714 |
|  | LDL cholesterol levels | 2.00E-11 | -0.0152177 | [0.011-0.02] | 32203549 |
|  | Phospholipids to total lipids ratio in very large VLDL | 1.00E-09 | -0.027556 | [0.019-0.036] | 35213538 |
|  | LDL (standard GWA) | 4.00E-08 | 0.014162 | [0.0091-0.0192] | 37106081 |
|  | LDL cholesterol levels | 4.00E-10 | 0.0143 | [0.0098-0.0188] | 34594039 |
| rs345780410 | Total cholesterol levels | 3.00E-16 | - | - | 34887591 |
|  | ***Red cell distribution width*** | 3.00E-08 | - | - | 30595370 |
|  | Total cholesterol levels | 8.00E-10 | 0.0149 | [0.01-0.02] | 33462484 |
| rs2700892 | ApoA1 levels | 2.00E-12 | 0.0138725 | [0.01-0.018] | 32203549 |
|  | HDL cholesterol levels | 4.00E-12 | -0.0174 | [0.013-0.022] | 33462484 |
|  | ApoA1 levels | 1.00E-09 | -0.0153 | [0.01-0.02] | 33462484 |
| rs12533280 | Non-HDL cholesterol levels | 7.00E-15 | 0.0187461 | [0.015-0.023] | 34887591 |
|  | LDL cholesterol levels | 2.00E-21 | - | - | 34887591 |
|  | LDL cholesterol levels | 8.00E-19 | 0.0180348 | [0.015-0.021] | 34887591 |
| rs2726111 | Triglycerides to total lipids ratio in large VLDL | 2.00E-08 | 0.0247447 | [0.016-0.033] | 35213538 |
| rs2700902 | ***High light scatter reticulocyte count*** | 6.00E-13 | -0.017078849 | [0.012-0.022] | 32888494 |
|  | ***High light scatter reticulocyte percentage of red cells*** | 1.00E-11 | -0.01606981 | [0.011-0.021] | 32888494 |
|  | ***Reticulocyte count*** | 5.00E-12 | -0.016427623 | [0.012-0.021] | 32888494 |
|  | ***Reticulocyte fraction of red cells*** | 1.00E-10 | -0.015298515 | [0.011-0.02] | 32888494 |
| rs11771125 | Non-HDL cholesterol levels | 3.00E-19 | - | - | 34887591 |
| rs4302748 | Platelet count | 5.00E-06 | - | - | 21507922 |
| rs12533197 | HDL cholesterol levels | 1.00E-08 | 0.0212412 | [0.014-0.029] | 35213538 |
|  | Cholesteryl ester levels in HDL | 1.00E-08 | 0.0215404 | [0.014-0.029] | 35213538 |
|  | HDL cholesterol levels | 2.00E-27 | - | - | 34887591 |
|  | HDL cholesterol levels | 3.00E-17 | -0.0154886 | [0.013-0.018] | 34887591 |
| rs66763009 | HDL cholesterol levels | 1.00E-16 | 0.0160456 | [0.012-0.02] | 32203549 |
| rs762848948 | Cholesteryl esters to total lipids ratio in small VLDL | 1.00E-08 | 0.0254458 | [0.017-0.034] | 35213538 |
| rs5883535 | Cholesterol levels in medium HDL | 4.00E-09 | 0.0226681 | [0.015-0.03] | 35213538 |
|  | Concentration of medium HDL particles | 7.00E-09 | 0.0222505 | [0.015-0.03] | 35213538 |
|  | HDL cholesterol levels | 6.00E-14 | -0.0161 | [0.012-0.02] | 34594039 |
|  | ApoA1 levels | 1.00E-08 | 0.0218948 | [0.014-0.029] | 35213538 |
|  | Total lipid levels in HDL | 2.00E-08 | 0.0209479 | [0.014-0.028] | 35213538 |
|  | Concentration of HDL particles | 1.00E-08 | 0.0222616 | [0.015-0.03] | 35213538 |
|  | Phospholipid levels in HDL | 4.00E-08 | 0.0206639 | [0.013-0.028] | 35213538 |
|  | Cholesteryl ester levels in medium HDL | 4.00E-09 | 0.0226713 | [0.015-0.03] | 35213538 |
|  | Free cholesterol levels in medium HDL | 6.00E-09 | 0.0220823 | [0.015-0.03] | 35213538 |
|  | Total lipid levels in medium HDL | 1.00E-08 | 0.0220118 | [0.014-0.03] | 35213538 |
|  | Phospholipid levels in medium HDL | 5.00E-08 | 0.0212757 | [0.014-0.029] | 35213538 |
| rs9648428 | Obesity-related traits | 3.00E-06 | 0.03 | [NR] | 23251661 |
| rs35580606 | HDL cholesterol levels | 1.00E-08 | 0.0108931 | [0.0071-0.0146] | 32203549 |
| rs10848 | ***High light scatter reticulocyte count*** | 6.00E-10 | -0.013847215 | [0.0095-0.0182] | 32888494 |
|  | ***Mean spheric corpuscular volume*** | 2.00E-12 | 0.015688576 | [0.011-0.02] | 32888494 |
|  | ***Reticulocyte count*** | 7.00E-10 | -0.013858353 | [0.0095-0.0183] | 32888494 |

**Table S2. The association of genetic variations of Eepd1 with NAFLD and liver fat are listed as follows.**

| ***EEPD1* SNPs** | **traits** | **Position and *p-*value** | **PubMed ID** |
| --- | --- | --- | --- |
| rs551923736 | Liver Fat | 7:36244028_TGAGGAGGAG/T P Value: 5.20 × 10^-4 | 34128465 |
| rs11771245 | NAFLD | 7:36115541_C/T P Value: 3.07 × 10^-3 Ref. Allele: C | 34841290 |
| rs2726069 | NAFLD | 7:36170392_G/T P Value: 4.61 × 10^-3 Ref. Allele: G | 31311600 |
| rs73104988 | NAFLD | 7:36142414_G/A P Value: 2.58 × 10^-3 Ref. Allele: G LD Reference Variant Show label rsID: rs73104988 | 32298765 |
| rs117240369 | NAFLD | 7:36016164_G/T P Value: 3.55 × 10^-5 Ref. Allele: G LD Reference Variant Show label rsID: rs117240369 | 34535985 |

**Table S3. The FDA-approved drugs in this assay are listed as follows.**

| **Number** | **Name** | **High content screening** |
| --- | --- | --- |
| 1 | Iloperidone | 1.276583977 |
| 2 | Dexrazoxane hydrochloride | 1.167410657 |
| 3 | Ciclopirox | 1.238401175 |
| 4 | Menthol | 1.33213592 |
| 5 | Clemizole hydrochloride | 1.172773634 |
| 6 | Canagliflozin | 1.160023773 |
| 7 | Zafirlukast | 1.180905973 |
| 8 | Desonide | 1.148418113 |
| 9 | Racecadotril | 1.118519866 |
| 10 | Betamethasone valerate | 1.256873414 |
| 11 | Prothionamide | 1.20012767 |
| 12 | Mebeverine hydrochloride | 1.125212164 |
| 13 | Nifedipine | 1.137074901 |
| 14 | Doxylamine succinate | 1.19476977 |
| 15 | Medroxyprogesterone Acetate | 1.029936911 |
| 16 | Isoniazid | 1.091240816 |
| 17 | Hesperidin | 1.144215057 |
| 18 | Pyridoxine | 1.202399476 |
| 19 | Terfenadine | 1.274455598 |
| 20 | Proglumide | 1.143120357 |
| 21 | Repaglinide | 1.252254915 |
| 22 | Prostaglandin E1 | 1.417996769 |
| 23 | Rosuvastatin calcium | 1.240801666 |
| 24 | Gestodene | 1.25038049 |
| 25 | Asiatic acid | 1.169875295 |
| 26 | 18β-Glycyrrhetinic acid | 1.239948811 |
| 27 | Cyclandelate | 1.123432251 |
| 28 | Vitamin B12 | 1.168533281 |
| 29 | Alcaftadine | 1.466581372 |
| 30 | Carprofen | 1.062854567 |
| 31 | Sulfogaiacol | 1.200170337 |
| 32 | Bephenium (hydroxynaphthoate) | 1.215121316 |
| 33 | Urapidil hydrochloride | 1.15007354 |
| 34 | Fingolimod hydrochloride | 1.14556703 |
| 35 | Acetohexamide | 1.096763624 |
| 36 | Pikamilone | 1.246631444 |
| 37 | Olanzapine | 1.063496527 |
| 38 | Pheniramine maleate | 1.292289685 |
| 39 | Benzydamine | 1.072633545 |
| 40 | Bicuculline | 1.044261161 |
| 41 | Magnolol | 1.334085134 |
| 42 | Lactulose | 1.236433605 |
| 43 | Etoricoxib | 1.311461197 |
| 44 | Olmesartan Medoxomil | 1.246256324 |
| 45 | Sacubitril/Valsartan | 1.15849088 |
| 46 | Diperodon hydrochloride | 1.247539072 |
| 47 | D-Sorbitol | 1.120402591 |
| 48 | Ethaverine hydrochloride | 1.31123634 |
| 49 | Fostamatinib | 1.085429882 |
| 50 | Catharanthine | 1.048871654 |
| 51 | Dehydroandrographolide | 1.154722111 |
| 52 | Formoterol fumarate | 1.152185417 |
| 53 | Sodium 4-phenylbutyrate | 1.180337533 |
| 54 | Alimemazine hemitartrate | 1.016143413 |
| 55 | Vinpocetine | 1.070989249 |
| 56 | Gastrodenol | 1.123762067 |
| 57 | Estradiol (cypionate) | 1.124508303 |
| 58 | Levetiracetam | 1.117042724 |
| 59 | Trelagliptin succinate | 1.115328812 |
| 60 | Mometasone furoate | 1.032605024 |
| 61 | Revaprazan hydrochloride | 1.060538407 |
| 62 | Nateglinide | 1.240936266 |
| 63 | Glibenclamide | 0.926111298 |
| 64 | Pantoprazole sodium | 1.008727979 |
| 65 | Actarit | 1.000599757 |
| 66 | Enarodustat | 1.257395584 |
| 67 | Conivaptan hydrochloride | 1.3358082 |
| 68 | Glipizide | 1.016396185 |
| 69 | Mitiglinide calcium hydrate | 0.889980201 |
| 70 | DL-Xylose | 0.923159499 |
| 71 | Entacapone | 0.922280078 |
| 72 | Itopride hydrochloride | 1.240171394 |
| 73 | Doxepin hydrochloride | 0.883603414 |
| 74 | Diphenidol hydrochloride | 0.95111369 |
| 75 | Metaraminol tartrate | 0.969513354 |
| 76 | Duloxetine hydrochloride | 1.039172253 |
| 77 | Conivaptan hydrochloride | 1.16616784 |
| 78 | Glipizide | 1.164712763 |
| 79 | Mitiglinide calcium hydrate | 1.074852926 |
| 80 | DL-Xylose | 0.876440691 |
| 81 | Entacapone | 0.9170747 |
| 82 | Itopride hydrochloride | 1.073572433 |
| 83 | Doxepin hydrochloride | 1.096784721 |
| 84 | Diphenidol hydrochloride | 0.872637153 |
| 85 | Metaraminol tartrate | 0.85174704 |
| 86 | Duloxetine hydrochloride | 1.301031655 |
| 87 | Methscopolamine | 1.234941613 |
| 88 | Naloxegol oxalate | 1.151270198 |
| 89 | Pargyline hydrochloride | 0.991288593 |
| 90 | Spironolactone | 1.083381735 |
| 91 | Dabigatran etexilate | 1.20685964 |
| 92 | Acetylcholine chloride | 1.01730529 |
| 93 | Telaprevir | 1.005194938 |
| 94 | Rivastigmine | 0.973259406 |
| 95 | Demecarium bromide | 1.101615434 |
| 96 | Fluorometholone Acetate | 1.071387691 |
| 97 | Berberine | 0.959803183 |
| 98 | α-Vitamin E | 0.895281553 |
| 99 | Daphnetin | 0.976115961 |
| 100 | Sodium Aescinate | 1.050535945 |
| 101 | Beclometasone | 1.088268539 |
| 102 | Clopidogrel hydrogen sulfate | 0.968123959 |
| 103 | Ipratropium bromide monohydrate | 1.30051397 |
| 104 | Amcinonide | 0.771451625 |
| 105 | Cilnidipine | 0.995024689 |
| 106 | Tinoridine hydrochloride | 1.224196496 |
| 107 | Piribedil | 0.84685107 |
| 108 | Irinotecan hydrochloride trihydrate | 1.229625995 |
| 109 | Ledipasvir | 1.227692083 |
| 110 | Dipyridamole | 1.175320202 |
| 111 | Pramipexole dihydrochloride hydrate | 1.090131305 |
| 112 | Lusutrombopag | 0.884619115 |
| 113 | Elvitegravir | 1.20164734 |
| 114 | Asunaprevir | 0.815667905 |
| 115 | Ombitasvir | 0.909949128 |
| 116 | Stiripentol | 1.396921354 |
| 117 | Drospirenone | 1.055486245 |
| 118 | Elbasvir | 0.925349407 |
| 119 | Fursultiamine | 1.235967186 |
| 120 | Balsalazide sodium hydrate | 0.932375256 |
| 121 | Perospirone | 0.9010124 |
| 122 | Grazoprevir | 1.23762936 |
| 123 | Tafamidis | 1.029403588 |
| 124 | Sulfaphenazole | 0.984298498 |
| 125 | Amylmetacresol | 1.143831938 |
| 126 | Sacubitril | 0.916848561 |
| 127 | Cyclothiazide | 0.937617061 |
| 128 | Lodoxamide | 0.87533904 |
| 129 | Indacaterol maleate | 1.047371086 |
| 130 | Ebastine | 1.273945373 |
| 131 | Beta-Sitosterol | 0.836176227 |
| 132 | Chlorotrianisene | 0.98594616 |
| 133 | Flumazenil | 1.256654855 |
| 134 | Cetirizine dihydrochloride | 0.950776171 |
| 135 | Aminohippurate sodium | 0.935868523 |
| 136 | Medroxyprogesterone | 1.034275082 |
| 137 | Odanacatib | 1.050625133 |
| 138 | Etofenamate | 1.124686773 |
| 139 | Olprinone | 1.320802331 |
| 140 | Nintedanibesylate | 1.05217073 |
| 141 | Flurbiprofen Axetil | 0.961008603 |
| 142 | Deoxycholic acid sodium salt | 1.041005741 |
| 143 | Histamine | 0.914912906 |
| 144 | Ethacrynic acid | 1.531839513 |
| 145 | Isopropamide Iodide | 0.979544177 |
| 146 | Sugammadex sodium | 1.218371545 |
| 147 | Bepridil hydrochloride | 1.24670536 |
| 148 | Fludrocortisone acetate | 0.946059975 |
| 149 | GUANABENZ | 1.070387441 |
| 150 | Triprolidine hydrochloride monohydrate | 0.940250215 |
| 151 | Diflorasone | 0.899535916 |
| 152 | AICAR | 1.093181586 |
| 153 | Irsogladine | 0.966077267 |
| 154 | Benazepril hydrochloride | 1.103843629 |
| 155 | Canrenone | 0.856372726 |
| 156 | Allantoin | 0.892349893 |
| 157 | Ipratropium Bromide | 1.2344248 |
| 158 | Bufexamac | 0.966288414 |
| 159 | Carbidopa | 1.383969251 |
| 160 | Guaiacol | 1.089941696 |
| 161 | 3-Pyridinemethanol | 0.998637833 |
| 162 | Alibendol | 1.213199687 |
| 163 | Malotilate | 1.094724567 |
| 164 | Gabapentin enacarbil | 1.016806129 |
| 165 | Lithocholic acid | 0.901022155 |
| 166 | Piperonyl butoxide | 0.97451346 |
| 167 | Propylthiouracil | 1.066162832 |
| 168 | Sivelestat | 1.249713286 |
| 169 | Sivelestat | 1.249713286 |
| 170 | Ripasudil | 1.380629319 |
| 171 | 4-Aminosalicylic acid | 0.932997576 |
| 172 | Lacidipine | 0.956611685 |
| 173 | Thioctamide | 1.195644729 |
| 174 | Glycyrrhizic acid | 1.28752089 |
| 175 | Sodium 2-mercaptoethanesulfonate | 1.139455778 |
| 176 | Fenbufen | 1.124669235 |
| 177 | Carbimazole | 0.978133357 |
| 178 | Penciclovir | 1.084567295 |
| 179 | Naftopidil | 1.067727926 |
| 180 | Aspartame | 1.507187753 |
| 181 | Mozavaptan | 1.291185872 |
| 182 | Voxilaprevir | 1.228197612 |
| 183 | Enalapril Maleate | 1.171221509 |
| 184 | Zaltoprofen | 1.282326629 |
| 185 | Butoconazole nitrate | 0.908545918 |
| 186 | Inosine | 1.331373971 |
| 187 | Osalmid | 0.996790883 |
| 188 | Terbinafine hydrochloride | 1.130076766 |
| 189 | Trimebutine | 1.244378368 |
| 190 | Valpromide | 1.384084691 |
| 191 | Naratriptan hydrochloride | 1.068161409 |
| 192 | Acefylline | 1.336463931 |
| 193 | Diphenmanilmethylsulfate | 1.403546223 |
| 194 | Ramosetron hydrochloride | 1.267212081 |
| 195 | Heptaminol hydrochloride | 1.241909605 |
| 196 | D-Mannitol | 1.168363239 |
| 197 | Cloxiquine | 1.106303872 |
| 198 | Flopropione | 1.095818739 |
| 199 | D-Glucuronic acid lactone | 1.137553957 |
| 200 | Uracil | 1.512693731 |
| 201 | Ambroxol hydrochloride | 1.178843747 |
| 202 | Piracetam | 1.653578653 |
| 203 | Catechin | 1.243752202 |
| 204 | Xylitol | 1.127690087 |
| 205 | Thiamine hydrochloride | 1.212881201 |
| 206 | Monobenzone | 0.976922171 |
| 207 | Aspirin | 1.105634282 |
| 208 | Salicylamide | 1.326427607 |
| 209 | L-Ascorbic acid | 1.231889988 |
| 210 | Docusate sodium | 1.16470773 |
| 211 | Sulfapyridine | 1.094120912 |
| 212 | Ethamsylate | 1.040062865 |
| 213 | Piperidolate hydrochloride | 1.549596357 |
| 214 | Betaine chloride | 1.268190336 |
| 215 | Methacholine Chloride | 1.167698137 |
| 216 | DL-Panthenol | 1.28617779 |
| 217 | Piperacetazine | 1.421996502 |
| 218 | Carglumic Acid | 1.307317474 |
| 219 | Theophylline monohydrate | 1.23372941 |
| 220 | Tolperisone hydrochloride | 1.162767238 |
| 221 | Glucosamine hydrochloride | 1.502302971 |
| 222 | Ifenprodil Tartrate | 1.106918302 |
| 223 | Paeonol | 1.285738576 |
| 224 | Isoprenaline hydrochloride | 1.531529097 |
| 225 | Beclamide | 1.250249413 |
| 226 | Lumacaftor | 1.660687808 |
| 227 | Betahistine mesylate | 1.12143851 |
| 228 | Famotidine | 1.330368239 |
| 229 | Artemether | 1.39989389 |
| 230 | Empagliflozin | 1.240543086 |
| 231 | Clorprenaline hydrochloride | 1.144733391 |
| 232 | Ipriflavone | 1.385383968 |
| 233 | Inositol nicotinate | 1.026145356 |
| 234 | Clonixin | 1.456183273 |
| 235 | Bifendate | 1.243617306 |
| 236 | D-Pantothenic acid hemicalcium salt | 1.142272117 |
| 237 | Sertraline hydrochloride | 1.009872106 |
| 238 | Sofalcone | 1.202353522 |
| 239 | Teprenone | 0.87641612 |
| 240 | Trimethobenzamide hydrochloride | 1.198248561 |
| 241 | Minaprine dihydrochloride | 1.115320784 |
| 242 | Eperisone hydrochloride | 1.040199371 |
| 243 | Acetyl-L-carnitine hydrochloride | 1.193315321 |
| 244 | Methylbenactyzium Bromide | 1.056747933 |
| 245 | Ampiroxicam | 1.417496341 |
| 246 | Cysteamine hydrochloride | 1.170017039 |
| 247 | Synephrine | 0.981607434 |
| 248 | Calcium dobesilate | 1.009876124 |
| 249 | Epinastine hydrochloride | 0.88429133 |
| 250 | Felypressin | 1.277921487 |
| 251 | Valethamate bromide | 1.383151041 |
| 252 | Trepibutone | 1.047426182 |
| 253 | Molsidomine | 1.178048019 |
| 254 | Mebhydrolin napadisylate | 0.979302679 |
| 255 | Suplatast (Tosilate) | 1.18883466 |
| 256 | Nitrendipine | 1.111798323 |
| 257 | Rocuronium bromide | 1.17044042 |
| 258 | Urapidil | 1.11921901 |
| 259 | Seratrodast | 0.964707283 |
| 260 | Levodropropizine | 1.191161314 |
| 261 | Bromisoval | 1.342706026 |
| 262 | Methyl-Hesperidin | 0.995091606 |
| 263 | Modaline sulfate | 1.018711676 |
| 264 | Buflomedil hydrochloride | 1.239538195 |
| 265 | Trometamol hydrochloride | 1.050155992 |
| 266 | Amiloride hydrochloride dihydrate | 1.086433496 |
| 267 | Estrone | 0.949721655 |
| 268 | Zolmitriptan | 1.058877361 |
| 269 | Penfluridol | 0.947864524 |
| 270 | Meclizine dihydrochloride | 1.4385486 |
| 271 | Thymalfasin | 1.258197501 |
| 272 | Oxiracetam | 1.432290838 |
| 273 | (±) Clopidogrel hydrogen sulfate | 1.248970311 |
| 274 | Nicardipine hydrochloride | 1.252297489 |
| 275 | Levosulpiride | 1.165832383 |
| 276 | Cromolyn sodium | 1.307423554 |
| 277 | Diacerein | 1.089470629 |
| 278 | Tanshinone I | 1.10861091 |
| 279 | Idarubicin hydrochloride | 19.50571002 |
| 280 | Betrixaban maleate | 1.209454297 |
| 281 | Scoparone | 1.336678906 |
| 282 | Benzthiazide | 1.143251746 |
| 283 | Clidinium bromide | 1.110952297 |
| 284 | Agomelatine | 1.120109771 |
| 285 | Trospium chloride | 1.156470451 |
| 286 | Trilostane | 1.110042516 |
| 287 | Feprazone | 1.07865854 |
| 288 | Sertindole | 1.017326546 |
| 289 | Tranilast | 0.897739539 |
| 290 | Pidotimod | 1.105046483 |
| 291 | Chlormadinone acetate | 1.130246011 |
| 292 | Silibinin | 1.163709718 |
| 293 | Allylestrenol | 1.103429536 |
| 294 | Flaconitine | 1.091204896 |
| 295 | Benactyzine hydrochloride | 1.033167342 |
| 296 | Topiroxostat | 1.782178682 |
| 297 | Brivudine | 1.246808091 |
| 298 | S-Adenosyl-L-Methionine | 1.086108641 |
| 299 | Indinavir sulfate | 0.879291994 |
| 300 | Cyclopenthiazide | 1.122714423 |
| 301 | Deserpidine | 1.408909726 |
| 302 | Fadrozole hydrochloride | 1.261003827 |
| 303 | Enoximone | 1.336964952 |
| 304 | Tranylcypromine hemisulfate | 1.067452615 |
| 305 | Pentetic Acid | 1.274279479 |
| 306 | L-Glutamic acid | 1.071031727 |
| 307 | Citric acid | 1.315379855 |
| 308 | Tiagabine hydrochloride | 1.434722824 |
| 309 | Dehydrocholate sodium | 1.110049769 |
| 310 | Warfarin sodium | 1.343920457 |
| 311 | L-Ascorbic acid sodium salt | 1.319598925 |
| 312 | Difamilast | 1.206990338 |
| 313 | Pazopanib Hydrochloride | 1.190595684 |
| 314 | Propagermanium | 1.065334217 |
| 315 | 10-Undecenoic acid | 1.231755333 |
| 316 | GestonoroneCapronate | 1.508183524 |
| 317 | Capsaicin | 0.916310522 |
| 318 | Bempedoic acid | 1.336994318 |
| 319 | Sofpironium bromide | 1.046287843 |
| 320 | Buformin hydrochloride | 1.20207542 |
| 321 | Triptolide | 1.163489542 |
| 322 | Dexrazoxane | 1.329283422 |
| 323 | Pimethixene | 1.169172573 |
| 324 | Oliceridine hydrochloride | 1.055822329 |
| 325 | Pericyazine | 1.094903434 |
| 326 | Prazosin | 1.21675833 |
| 327 | LumateperoneTosylate | 1.013980094 |
| 328 | Levamlodipine | 1.188548602 |
| 329 | Afloqualone | 1.009957923 |
| 330 | Sarpogrelate hydrochloride | 1.164488792 |
| 331 | Taurochenodeoxycholic Acid | 1.147241582 |
| 332 | L-Ornithine hydrochloride | 1.097601774 |
| 333 | Propyl gallate | 0.913328064 |
| 334 | Lappaconitine hydrobromide | 1.174784151 |
| 335 | Melitracen hydrochloride | 1.095090454 |
| 336 | Lorcaserin HCl | 1.073168057 |
| 337 | 7-Dehydrocholesterol | 0.993710369 |
| 338 | Thiamine pyrophosphate | 1.272402637 |
| 339 | Squalene | 1.229557227 |
| 340 | 1-Hexadecanol | 1.423722009 |
| 341 | Diroximel fumarate | 1.157406757 |
| 342 | Ameziniummethylsulfate | 1.15010184 |
| 343 | Diflucortolone valerate | 1.330555625 |
| 344 | Midodrine | 0.989406044 |
| 345 | Solifenacin | 1.077923831 |
| 346 | Guanabenz hydrochloride | 1.017093074 |
| 347 | Cromoglicic acid | 0.986522928 |
| 348 | Bromodiphenhydramine hydrochloride | 1.158185327 |
| 349 | Methyclothiazide | 0.945678553 |
| 350 | (-)-Securinine | 1.046994941 |
| 351 | Acetylcholine bromide | 0.985978252 |
| 352 | Estramustine | 1.036347067 |
| 353 | Vitamin D3 | 0.935837402 |
| 354 | hydrocotarnine | 1.050931919 |
| 355 | Rutin hydrate | 0.901485633 |
| 356 | Diclofenac Epolamine | 0.878061963 |
| 357 | Ifenprodil | 1.012440782 |
| 358 | Evocalcet | 0.917345586 |
| 359 | Calcifediol monohydrate | 0.980888321 |
| 360 | Fesoterodine | 1.103028932 |
| 361 | Dextromethorphan | 1.101089593 |
| 362 | Nedocromil | 0.891559249 |
| 363 | Gefarnate | 1.103280501 |
| 364 | Eicosapentaenoic Acid | 1.116560545 |
| 365 | Levamlodipine besylate | 1.040044309 |
| 366 | Sitagliptin phosphate | 0.935314728 |
| 367 | Indigo | 0.93761441 |
| 368 | α-Lipoic Acid | 0.899054249 |
| 369 | Palmitic acid | 0.952791515 |
| 370 | Daidzein | 1.079370333 |
| 371 | Perphenazine | 0.91479039 |
| 372 | Ganirelix Acetate | 0.93996543 |
| 373 | Vidarabine phosphate | 0.928061128 |
| 374 | Icatibant Acetate | 1.071604104 |
| 375 | Terlipressin Acetate | 1.157613781 |
| 376 | Palmatine | 1.182768606 |
| 377 | Pentamidine dihydrochloride | 1.513419172 |
| 378 | Dexamethasone Phosphate disodium | 1.046070384 |
| 379 | Thiamine nitrate | 0.974696895 |
| 380 | Resorcinol monoacetate | 1.000061629 |
| 381 | Feprazone | 0.831360192 |
| 382 | Doxapram hydrochloride hydrate | 0.964223062 |
| 383 | Sertindole | 1.07392283 |
| 384 | Proxyphylline | 1.105414464 |
| 385 | Levomefolic Acid | 1.352814971 |
| 386 | Melperone | 1.729931516 |
| 387 | Efonidipine hydrochloride monoethanolate | 0.899491137 |
| 388 | Ulipristal | 0.984473582 |
| 389 | Metoclopramide hydrochloride hydrate | 0.976135314 |
| 390 | (±)-Carnitine chloride | 0.97079936 |
| 391 | Lasmiditan succinate | 1.061452836 |
| 392 | Bromfenac Sodium | 1.264088939 |
| 393 | Avacopan | 0.823233434 |
| 394 | L-Histidine | 1.003121813 |
| 395 | Choline | 1.090056662 |
| 396 | Hydroxyzine | 1.210048397 |
| 397 | Retigabine dihydrochloride | 1.869126079 |
| 398 | Tacrine hydrochloride (hydrate) | 0.844663531 |
| 399 | Tenapanor | 1.144500381 |
| 400 | Scopolamine hydrobromide | 1.100189318 |
| 401 | Maribavir | 0.970151825 |
| 402 | Dipotassium glycyrrhizinate | 1.186251749 |
| 403 | Decloxizine dihydrochloride | 0.977877748 |
| 404 | Zinc sulfate heptahydrate | 0.959665174 |
| 405 | Fostemsavir | 1.235065144 |
| 406 | Laurocapram | 0.854951661 |
| 407 | Sucrose | 0.98602996 |
| 408 | Icotinib Hydrochloride | 0.945407806 |
| 409 | Ertugliflozin L-pyroglutamic acid | 0.92183302 |
| 410 | L-Alanine | 0.856957354 |
| 411 | L-Histidine | 0.87421335 |
| 412 | Lisinopril dihydrate | 1.178285832 |
| 413 | Pemirolast potassium | 0.843815738 |
| 414 | DL-Methionine | 1.148141289 |
| 415 | Amikacin sulfate | 1.042477773 |

| **Table S4. The antibodies，critical commercial assays kits and chemicals are listed as follows.** |
| --- |

| **Antibodies** | | | |
| --- | --- | --- | --- |
| Rabbit anti-beta actin | Abclonal | Cat# AC038; RRID: AB_2863784 |  |
| Rabbit anti-Phospho-ULK1 | Abclonal | Cat# AP0760; RRID: AB_2771636 |  |
| Rabbit anti-ULK1 | Abclonal | Cat# A8529; RRID: AB_2772810 |  |
| Rabbit anti-TGF-beta1 | Abclonal | Cat# A2124; RRID: AB_2764143 |  |
| Rabbit anti-alpha smooth muscle actin | Abcam | Cat# Ab5694; RRID: AB_2223021 |  |
| Rabbit anti-collagen IV antibody | Abcam | Cat# Ab6586 |  |
| Rabbit anti-EEPD1 antibody | Abcam | Cat# Ab220501 |  |
| Rabbit anti-Hif1-alpha | Cell Signaling Technology | Cat# 36169S |  |
| Rabbit anti-Phospho-Histone H2A.X (Ser139) | Cell Signaling Technology | Cat# 2577S |  |
| Rabbit anti-SQSTM1/p62 Antibody | Cell Signaling Technology | Cat# 39749S |  |
| Rabbit anti-LC3 Antibody | Cell Signaling Technology | Cat# 12741S |  |
| Mouse anti-Ubiquitin | Cell Signaling Technology | Cat# 3936S |  |
| Rabbit anti-p38 MAPK | Cell Signaling Technology | Cat# 8690S |  |
| Rabbit anti-Phospho-p38 MAPK (Thr180/Tyr182) | Cell Signaling Technology | Cat# 4511S |  |
| Rabbit anti-Phospho-SAPK/JNK (Thr183/Tyr185) | Cell Signaling Technology | Cat# 4668S |  |
| Rabbit anti-JNK Antibody | Cell Signaling Technology | Cat# 9252S |  |
| Peroxidase AffiniPure Goat Anti-Rabbit IgG (H+L) | Jackson ImmunoResearch | Cat# Fs111-035-003;  RRID: AB_2313567 |  |
| Peroxidase AffiniPure Goat Anti-Mouse IgG(H+L) | Jackson ImmunoResearch | Cat# Fs115-035-003;  RRID: AB_10015289 |  |
| Rabbit anti-F4/80 Polyclonal antibody | Proteintech | Cat# 28463-1-AP;  RRID: AB_2881149 |  |
| Rabbit anti-alpha actinin polyclonal antibody | Proteintech | Cat# 11313-2-AP;  RRID: AB_2223815 |  |
| Rabbit anti-fibronectin polyclonal antibody | Proteintech | Cat# 15613-1-AP;  RRID: AB_2105691 |  |
| Rabbit anti-CTGF polyclonal antibody | Proteintech | Cat# 25474-1-AP;  RRID: AB_2918089 |  |
| Anti-FLAG-HRP | Sigma-Aldrich | Cat# F7425 |  |

| **Critical commercial assays** | | |
| --- | --- | --- |
| BCA Assay kit | ThermoFisher Scientific | Cat# 23225 |
| Passive Lysis Buffer, 5X | Promega | Cat# E1941 |
| TG Assay kit | Applygene | Cat# E1013-105 |
| AST kit | NJJCBIO | Cat# C010-2-1 |
| ALT kit | NJJCBIO | Cat# C009-2-1 |
| 5X ABScript III RT Mix | Abclonal | Cat# RM21478 |
| SYBR Green PCR Master Mix | Vazyme, China | Cat# Q511-02 |
| Triton-100 vetec reagent grade | Sigma-Aldrich | Cat# V900502 |
| Goat anti-Rabbit IgG (H+L) Secondary Antibody, Alexa Fluor 568 | ThermoFisher Scientific | Cat# A-11011 |
| Lipofectamine 3000 Transfection Kit | Invitrogen | Cat# L3000-015 |

**Chemicals, peptides, and recombinant proteins**

| Dulbecco's modified Eagle's medium (DMEM) | ThermoFisher Scientific | Cat# C11995500BT |
| --- | --- | --- |
| Penicillin/Streptomycin | ThermoFisher Scientific | Cat# 15140-122 |
| protein A/G plus agarose beads | Beyotime | Cat# P2055 |
| Anti-DYKDDDDK Affinity Beads | Smart-Lifesciences | Cat# SA042025 |
| TRIzol reagent | ThermoFisher Scientific | Cat# 15596 |
| Lipofectamine RNAiMAX Reagent | Invitrogen | Cat# 13778-150 |
| Skim milk powder | BioFroxx | Cat# 1172GR500 |
| Bovine Serum Albumin(BSA) | Sigma-Aldrich | Cat# A8806 |
| Sodium palmitate | Sigma-Aldrich | Cat# P9767 |
| Fetal Bovine Serum | ThermoFisher Scientific | Cat#16000-044 |
| Collagenase type I | Worthington biochemical corporation | Cat# 4196 |
| Rat Collagen I | R&D Systems | Cat# 3440-005-01 |
| PERCOLL | BIODEE | Cat# DE-17-0891-02G |
| Phosphate Buffer Saline（1X） | Yuanxiang Medical | Cat# YXB5001 |
| Phosphatase inhibitor cocktail 1（100X in DMSO) | Apexbio | Cat# K1015-A |
| Phosphatase inhibitor cocktail 2（100X in ddH2O) | Apexbio | Cat# K1015-B |
| Protease Inhibitor Cocktail | Sigma-Aldrich | Cat# S8830 |
| SDS-PAGE Loading Buffer (non-Reducing,5x) | Cwbiotech | Cat# CW0028S |
| Proteinase K Solution | ThermoFisher Scientific | Cat# AM2548 |
| Oleic acid | Sigma-Aldrich | Cat# O1008 |
| Palmitic acid | Sigma-Aldrich | Cat# P0500 |
| Anti-fluorescence quenching mounting solution | Beyotime | Cat# P0131 |
| Polybrene | Sigma-Aldrich | N/A |

| **Table S5. The primers used in the qRT-PCR are listed as follows.** | | |
| --- | --- | --- |
| IL-6-F (mouse) | TAGTCCTTCCTACCCCAATTTCC |  |
| IL-6-R (mouse) | TTGGTCCTTAGCCACTCCTTC |  |
| IL1β-F (mouse) | GCAACTGTTCCTGAACTCAACT |  |
| IL1β-R (mouse) | ATCTTTTGGGGTCCGTCAACT |  |
| IL10-F (mouse) | GCTCTTACTGACTGGCATGAG |  |
| IL10-R (mouse) | CGCAGCTCTAGGAGCATGTG |  |
| TNFα-F (mouse) | CCCTCACACTCAGATCATCTTCT |  |
| TNFα-R (mouse) | GCTACGACGTGGGCTACAG |  |
| Tgf-β1-F (mouse) | CTCCCGTGGCTTCTAGTGC |  |
| Tgf-β1-R (mouse) | GCCTTAGTTTGGACAGGATCTG |  |
| Atg10-F (mouse) | GTAGTTACCAAGTGCCGGTTC |  |
| Atg10-R (mouse) | AGCTAACGGTCTCCCATCTAAA |  |
| Atg5-F (mouse) | AGCCAGGTGATGATTCACGG |  |
| Atg5-R (mouse) | GGCTGGGGGACAATGCTAA |  |
| Atg9a-F (mouse) | CAGTTTGACACTGAATACCAGCG |  |
| Atg9a-R (mouse) | AATGTGGTGCCAAGGTGATTT |  |
| Wipi1-F (mouse) | CTGCTTCTCTTTCAACCAAGACT |  |
| Wipi1-R (mouse) | ACGTCAGGGATTTCATTGCTT |  |
| Atg16l2-F (mouse) | GGAGAGACTCAGTCCAAGGAA |  |
| Atg16l2-R (mouse) | CCACGTCATTGCAGTAGGAAAG |  |
| Pik3c3-F (mouse) | CCTGGACATCAACGTGCAG |  |
| Pik3c3-R (mouse) | TGTCTCTTGGTATAGCCCAGAAA |  |
| Sqstm1-F (mouse) | ATGTGGAACATGGAGGGAAGA |  |
| Sqstm1-R (mouse) | GGAGTTCACCTGTAGATGGGT |  |
| Optn-F (mouse) | ATGTCCCATCAACCTCTGAGC |  |
| Optn-R (mouse) | TCAAATCGCCCTTTCATAGCTTG |  |
| Nbr1-F (mouse) | GGAAATCAGCTACAGATGCAAGT |  |
| Nbr1-R (mouse) | ATCCCAAGACTCTCACCAGTG |  |
| Ndp52-F (mouse) | GCCCCATACCTACCTTGCTG |  |
| Ndp52-R (mouse) | TCGAGGGATGAACTTTTCAGTG |  |
| Tax1bp1-F (mouse) | TGCACACTTGGAGTGCCATTA |  |
| Tax1bp1-R (mouse) | TGTTCAGGCATAGGAGACCATAA |  |
| Trim33-F (mouse) | AGGAAATGTGAACGTCTTCTGC |  |
| Trim33-R (mouse) | GGGATTTGGTAATGCTGGGAA |  |
| Trim24-F (mouse) | CGAATGAAACTCATGCAACAACA |  |
| Trim24-R (mouse) | AGGTGCCGTAACCTGTATGTAA |  |
| Trim37-F (mouse) | TCCAAGCTCTGTTGTTTCAGC |  |
| Trim37-R (mouse) | TTCCGCCCAACGACAGTTC |  |
| Trim68-F (mouse) | TCCCAGAACTTGAGCTACACC |  |
| Trim68-R (mouse) | AGACGGACCTTGTCTACAACA |  |
| Cd11b-F (mouse) | ATGGACGCTGATGGCAATACC |  |
| Cd11b-R (mouse) | TCCCCATTCACGTCTCCCA |  |
| Cd11c-F (mouse) | CTGGATAGCCTTTCTTCTGCTG |  |
| Cd11c-R (mouse) | GCACACTGTGTCCGAACTCA |  |
| Ly6c2-F (mouse) | TTGATTGAGGGTAAGTTCCCTGT |  |
| Ly6c2-R (mouse) | CCAGACATGACGTTTTCTCCAAA |  |
| Col3a1-F (mouse) | CTGTAACATGGAAACTGGGGAAA |  |
| Col3a1-R (mouse) | CCATAGCTGAACTGAAAACCACC |  |
| Col4a1-F (mouse) | CTGGCACAAAAGGGACGAG |  |
| Col4a1-R (mouse) | ACGTGGCCGAGAATTTCACC |  |
| Mmp2-F (mouse) | CAAGTTCCCCGGCGATGTC |  |
| Mmp2-R (mouse) | TTCTGGTCAAGGTCACCTGTC |  |
| 18s-F (mouse) | CGCCGCTAGAGGTGAAATTCT |  |
| 18s-R (mouse) | CATTCTTGGCAAATGCTTTCG |  |
| Acta2-F (mouse) | GTCCCAGACATCAGGGAGTAA |  |
| Acta2-R (mouse) | TCGGATACTTCAGCGTCAGGA |  |
| 36b4-F (mouse) | AGATTCGGGATATGCTGTTGGC |  |
| 36b4-R (mouse) | TCGGGTCCTAGACCAGTGTTC |  |
| Ctgf-F (mouse) | GGGCCTCTTCTGCGATTTC |  |
| Ctgf-R (mouse) | ATCCAGGCAAGTGCATTGGTA |  |
| Col1a1-F (mouse) | GCTCCTCTTAGGGGCCACT |  |
| Col1a1-R (mouse) | CCACGTCTCACCATTGGGG |  |
| Fibronectin-F (mouse) | ATGTGGACCCCTCCTGATAGT |  |
| Fibronectin-R (mouse) | GCCCAGTGATTTCAGCAAAGG |  |
| F4/80-F (mouse) | TGACTCACCTTGTGGTCCTAA |  |
| F4/80-R (mouse) | CTTCCCAGAATCCAGTCTTTCC |  |
| Nos2-F (mouse) | GTTCTCAGCCCAACAATACAAGA |  |
| Nos2-R (mouse) | GTGGACGGGTCGATGTCAC |  |
| Srebp1c-F (mouse) | TGACCCGGCTATTCCGTGA |  |
| Srebp1c-R (mouse) | CTGGGCTGAGCAATACAGTTC |  |
| Fasn-F (mouse) | GGAGGTGGTGATAGCCGGTAT |  |
| Fasn-R (mouse) | TGGGTAATCCATAGAGCCCAG |  |
| Acc1-F (mouse) | ATGGGCGGAATGGTCTCTTTC |  |
| Acc1-R (mouse) | TGGGGACCTTGTCTTCATCAT |  |
| Fabp1-F (mouse) | ATGAACTTCTCCGGCAAGTACC |  |
| Fabp1-R (mouse) | CTGACACCCCCTTGATGTCC |  |
| Fatp2-F (mouse) | TCCTCCAAGATGTGCGGTACT |  |
| Fatp2-R (mouse) | TAGGTGAGCGTCTCGTCTCG |  |
| Ccl2-F (mouse) | TTAAAAACCTGGATCGGAACCAA |  |
| Ccl2-R (mouse) | GCATTAGCTTCAGATTTACGGGT |  |
| p53-F (mouse) | CTCTCCCCCGCAAAAGAAAAA |  |
| p53-R (mouse) | CGGAACATCTCGAAGCGTTTA |  |
| p27-F (mouse) | TCAAACGTGAGAGTGTCTAACG |  |
| p27-R (mouse) | CCGGGCCGAAGAGATTTCTG |  |
| Parp1-F (mouse) | GGCAGCCTGATGTTGAGGT |  |
| Parp1-R (mouse) | GCGTACTCCGCTAAAAAGTCAC |  |
| Brca1-F (mouse) | CGAATCTGAGTCCCCTAAAGAGC |  |
| Brca1-R (mouse) | AAGCAACTTGACCTTGGGGTA |  |
| Ctip-F (mouse) | AATGGTCAACAGGATCAAGTAGC |  |
| Ctip-R (mouse) | GTAGCCGGTTAATGCCAGAAAA |  |
| Exo1-F (mouse) | TGGCTGTGGATACCTACTGTT |  |
| Exo1-R (mouse) | ATCGGCTTGACCCCATAAGAC |  |
| Dna2-F (mouse) | GGGTGGAGCTACTTCGGAAGA |  |
| Dna2-R (mouse) | CTCCTCGGCTCAGAACTGTCT |  |
| Cxcl1-F (mouse) | CTGGGATTCACCTCAAGAACATC |  |
| Cxcl1-R (mouse) | CAGGGTCAAGGCAAGCCTC |  |
